# Tuning Two-Photon Absorption in Rhodopsin Chromophore via Backbone Modification: The Story Told by CC2 and TD-DFT

**DOI:** 10.1021/acs.jctc.4c00675

**Published:** 2024-09-13

**Authors:** Saruti Sirimatayanant, Tadeusz Andruniów

**Affiliations:** Institute of Advanced Materials, Department of Chemistry, Wroclaw University of Science and Technology, Wyb. Wyspiańskiego 27, Wrocław 50-370, Poland

## Abstract

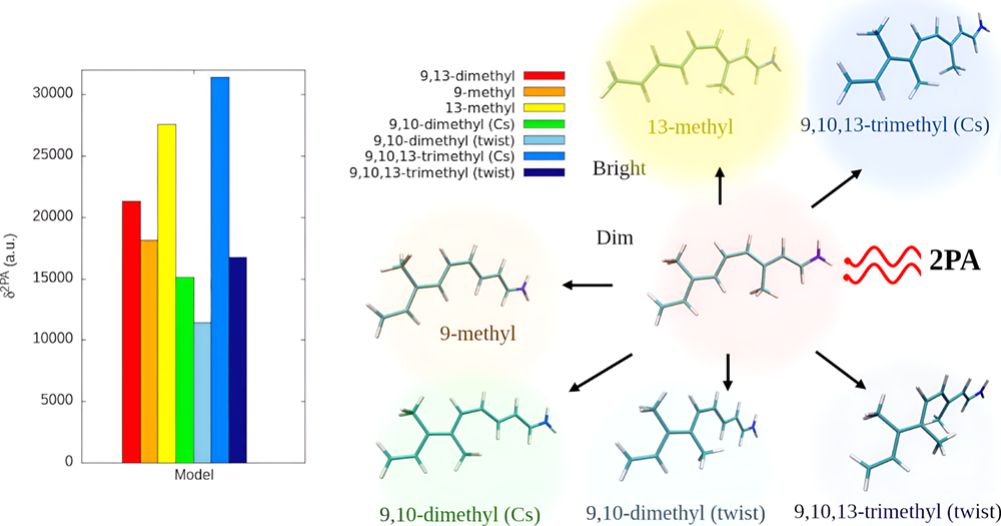

We investigate here a systematic way to tune two-photon transition strengths (δ^2PA^) and two-photon absorption (2PA) cross sections (σ^2PA^) of the rhodopsin’s chromophore 11-*cis*-retinal protonated Schiff base (RPSB) via the modulation of the methyl groups pattern along its polyene chain. Our team employed the resolution of identity, coupled cluster approximate second order (RI-CC2) method with Dunning’s aug-cc-pVDZ basis set, to determine the structural impact on δ^2PA^, as well as its correlation to both transition dipole moments and permanent electric dipole moments. Seven structures were probed in vacuo, including five-double-bond-conjugated model of the native chromophore, shortened by the β-ionone ring (RPSB5), and its de/methylated analogues: 9-methyl, 13-methyl, planar and twisted models of 9,10-dimethyl and 9,10,13-trimethyl. Our results demonstrate that the magnitude of δ^2PA^ is dictated by both the position and number of methylated groups attached to its polyene chain as well as the degree of dihedral twist that is introduced due to the de/methylation. In fact, a strong correlation between δ^2PA^ enhancement and the presence of a C13-methyl group in the planar RPSB5 species is found. Trends in δ^2PA^ values follow the trends observed in their corresponding changes in the permanent dipole moment upon the S0–S1 excitation nearly exactly. The assessment of four DFT functionals, i.e., M11, MN15, CAM-B3LYP, and BHandHLYP, previously found most successful in predicting 2PA properties in biological chromophores, points to a long-range-corrected hybrid meta-GGA M11 as the top-performing functional, albeit still delivering underestimated δ^2PA^ and σ^2PA^ values by a factor of 3.3–5.3 with respect to the CC2 results. In the case of global-hybrid meta-NGA (MN15), as well as CAM-B3LYP and BHandHLYP functionals, this factor deteriorates significantly to 6.7–20.9 and is mostly related to significantly lower quality of the ground- and excited-state dipole moments.

## Introduction

The VII helical transmembrane protein rhodopsin is a light-transducing protein found in the retina, specifically within the rod cells of vertebrates, and is responsible for dim light vision. At the heart of the rhodopsin protein lies its chromophore 11-*cis*-retinal covalently attached via the protonated Schiff base (RPSB) to the protein cavity’s ϵ-amine group of Lys296.^1,2^ Upon photoactivation through the absorption of a photon at 498 nm, the retinal photoisomerizes from the 11-*cis* configuration to the all-trans isomer and steers the protein conformational change,^3^ which in turn activates the G-Protein Coupled Receptor cascade, which leads to the scotopic visual signal in the brain.

Computational studies on rhodopsin and various other photoactive proteins from the opsin family have been demonstrated to nicely complement experimental studies.^4^ Computational methods can provide high quality electronic properties and geometries that may offer insights to the absorption or excitation properties of rhodopsin, as well as, detailed mechanisms of the photoisomerization pathway that may be difficult to elucidate experimentally.^4−9^ It has been found that the rhodopsin binding pocket is flexible enough to house structurally modified retinal chromophore to form artificial visual pigments.^10−14^ Some of these artificial pigments differ from the native rhodopsin only by deletion or insertion of methyl groups along the polyene chain of the chromophore. The substitutions of methyl groups directly affect the excited state lifetime, photoisomerization efficiency (quantum yield), and thermodynamic stability of rhodopsin’s photointermediates due to the tight and specific interactions between the chromophore and the closest amino acid side chains of the protein.^5,12,15−19^

One-photon absorption (1PA) spectroscopy of RPSB analogues has been assessed by many ab initio quantum chemical methods.^20−22^ The CC2 method was employed in multiple studies on optical properties of various photoactive proteins’ chromophores due to its ability to converge results to, and at a fraction of the cost of, higher level multireference methods.^22−24^ In a benchmark study from Walczak et al.,^22^ CC2 was among the different quantum methods investigated and was validated against CASPT2 for several de/methylated variants of RPSB5 models. CC2 showed great agreement with the reference, producing a mean absolute error (MAE) of all models at only 0.02 eV, which is also in line with the findings of Aquino et al.^25^ Oscillator strengths also displayed fairly agreeable results to the CASSCF (wave function)/CASPT2 (energies) reference, with a deviation of 0.01 up to 0.16, depending on the structure. The team also noted that oscillator strengths are sensitive to the degree of twist within the polyene chain. As compared with their planar counterparts, twisted structures may dampen oscillator strengths by up to 0.20. On the other hand, CC2 dipole moments are underestimated by nearly half the CASPT2 reference. For the native retinal model, change in dipole moments from the ground to the lowest-lying excited state goes from 9.53 to 5.40 D, when going from CASPT2 to CC2 results.

In recent years 2PA and two-photon microscopy (2PM) have gained increasing attention in various applications, ranging from 3D optical storage, optogenetics tools, in vivo imaging, and fluorescence spectroscopy.^26−30^ There is currently a high demand in rational design for optogenetic tools, among them rhodopsins, absorbing in the near-infrared region (NIR). One of the proposed gateways to reach NIR could include two-photon spectroscopy.^26^ While 2PA spectroscopy of rhodopsin and its modulation via mutations has been the topic of several computational studies,^6,31^ to the best of our knowledge, there have been no attempts to investigate the impact of RPSB chromophore backbone modification on its 2PA properties.

**Figure 1:**
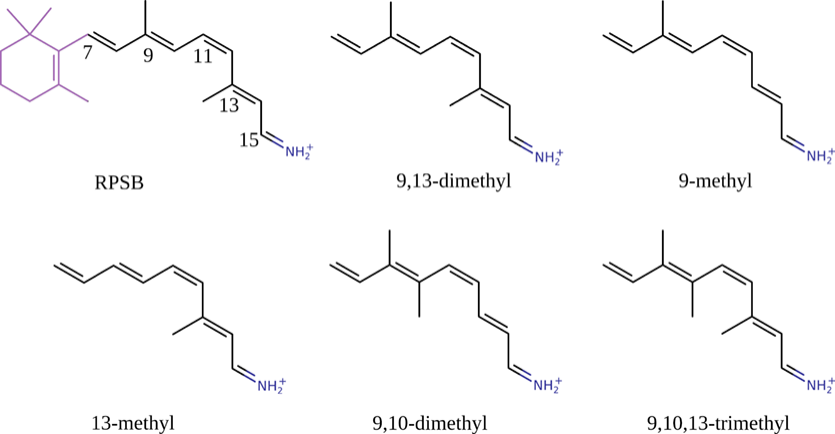
Chemical structure of the native RPSB chromophore along with structure of the truncated (RPSB5) native model (9,13-dimethyl) and its de/methylated analogues. 9,10-dimethyl and 9,10,13-trimethyl in both planar (Cs) and twisted conformations are considered. The atom numbering for the native RPSB is used for all investigated models.

Here, we investigate two-photon transition strengths of the native rhodopsin’s chromophore model RPSB5 that is truncated at the β-ionone ring and comprises the complete polyene chain with its protonated Schiff base, 5 double bonds, and 2 methyl constituents (9,13-dimethyl). In a femtosecond spectroscopy experiment by Wang et al.,^12^ the ultrafast photoisomerization mechanism was attributed to the nonbonding interactions between C13 methyl and C10 hydrogen, this sparked great attention to the investigation of these carbon positions for modulation of both excited state lifetime and photoisomerization pathway.^5,8,20^ Therefore, the 4 analogues also investigated here are based on de/methylation of the C9, C10, and C13 positions of the polyene chain (9-methyl, 13-methyl, 9,10-dimethyl, and 9,10,13-trimethyl) (see Figure 1). As noted by one of us,^22^ the planarity of analogues 9,10-dimethyl and 9,10,13-trimethyl is disturbed by steric hindrance due to the close proximity of neighboring methyl groups. Additionally, geometry optimization of the ground state equilibrium structure displayed nonplanar structures of these two analogues as global minima. Therefore, two sets of these structures were investigated: planar (Cs) and twisted (twist).

Our previous study^32^ benchmarked CC2 methodology against the approximately coupled cluster singles doubles and triples (CC3) for 2PA properties of reduced RPSB models, truncated to include only 3 (RPSB3) or 4 (RPSB4) of the conjugated double bonds. Vertical excitation energy of CC2 demonstrated convergence to CC3 with a deviation of only 0.019 eV, and a δ^2PA^ deviation of 4–11%, depending on the basis set.^32^ For these reasons, CC2 seems to be a promising method for investigating 2PA properties of rhodopsin’s chromophore. Therefore, we use here the RI-CC2 method with Dunning’s aug-cc-pVDZ^33^ basis set to investigate vertical excitation energies, two-photon transition strengths and 2PA cross sections, and dipole moments, of five different RPSB5 models with various de/methylation patterns; for two of them (9,10-dimethyl and 9,10,13-trimethyl) both the planar and twisted conformations were studied. Our first goal is to determine the impact of each methyl group along the polyene chain of RPSB5 on the 2PA properties to establish a rational design process for generating retinals with desired 2PA properties. The second goal is to evaluate how selected global-hybrid meta-NGA and range-separated hybrid meta-GGA functionals, MN15 and M11, respectively, perform in predicting two-photon absorption properties of rhodopsin chromophore’s analogues in relation to the results from the CC2 method, as well as the results obtained with CAM-B3LYP and BHandHLYP functionals. CAM-B3LYP and BHandHLYP performed best in the calculations of 2PA properties for RPSB3 and RPSB4,^32^ whereas MN15 and MN11 proved to be superior to other DFT functionals in describing 2PA of selected organic molecules.^34–36^

## Methodology

### Models and Geometries

The ground state geometry of the native RPSB5 and its analogues were adapted from Walczak et al.^22^ who performed geometry optimization at the CASPT2 level with an active space of 10 electrons in 10 π-orbitals with the Atomic Natural Orbital (ANO-L-VDZP) basis set. Of the 7 structures examined, 9,10-dimethyl (twist) and 9,10,13-trimethyl (twist) exhibit torsional deformation around C10–C11 and C11=C12 bonds, respectively, −167.9° and +9.9° for the former and −159.6° and +27.0° for the latter structure. While for 9,10-dimethyl (twist) the twisted structure is only 0.03 kcal/mol more stable than its planar counterpart at the CASPT2/ANO-L-VDZP level of theory, for 9,10,13-trimethyl (twist) this difference raises to as much as 9.31 kcal/mol.^22^

### Energies and Electronic Properties

All spectral properties, including one-photon excitation energies and transition dipole moments, two-photon transition strengths, and change in permanent electric dipole moments, are calculated using RI-CC2,^37,38^ and Time-Dependent DFT (TD-DFT) with the global-hybrid GGA functional BHandHLYP (50%),^39^ range-separated hybrid GGA functional CAM-B3LYP (19–65%),^40^ global-hybrid meta-nonseparable generalized approximation (meta-NGA) functional MN15 (44%),^41^ and range-separated hybrid meta-GGA (meta-GGA) functional M11 (44–100%).^42^ In parentheses, there are contributions from the HF-exchange energy to the DFT functional. The RI-CC2 and (TD)-DFT calculations with MN15 and M11 functionals, both taken from LibXC 5.0.0 library,^43^ were done with the Turbomole 2020 version 7.6.0^44,45^ while (TD)-DFT with CAM-B3LYP and BHandHLYP with Dalton2020 software.^46,47^ Aug-cc-pVDZ basis set was employed in RI-CC2 and TD-DFT calculations. In the case of M11 and MN15 functionals, 1PA properties were computed using gauge-invariant current-dependent formalism, which has not been implemented yet for 2PA properties, and thus δ^2PA^ are gauge-variant. 1PA and 2PA properties were obtained for the 5 lowest excited states; however, the discussion here will be limited to the properties of the S1 state, as for the higher-lying excited states the 2PA properties are either unrecoverable at the CC2 level of theory or suffer from the resonance enhancement with the S1 state at both CC2 and TD-DFT level of theories (see Tables S4–S8 in the Supporting Information). 2PA values are calculated with the quadratic response function,^48^ and represented in microscopic δ^2PA^ transition strengths and macroscopic σ^2PA^ cross sections. The conversion between microscopic and macroscopic values is discussed below.

To reduce the computational cost, for all RI-CC2 calculations, a total of 11 core orbitals for the single methylated structures (9-methyl and 13-methyl), 12 core orbitals for the doubly methylated structures (9,13-dimethyl and 9,10-dimethyl), and 13 core orbitals for triply methylated structures (9,10,13-trimethyl) were frozen. RI-CC2 calculations were verified with diagnostic tool %T2 for doubly excitation contributions.

Transition dipole moments (μ_01_) were obtained with the linear response function^49^ in length representation. Changes in dipole moments (Δμ) are calculated as , where “a” represents the Cartesian coordinates in *x*, *y*, and *z*. μ_00_ and μ_11_ represent permanent dipole moments in the ground state and first excited state, respectively. For simplicity, we denote , , , and .

### Conversion of Microscopic Two-Photon Transition Strengths to Macroscopic Two-Photon Absorption Cross Sections

The conversion between the two-photon transition moment in atomic units, readily given by the utilized software, into two-photon absorption cross-section in macroscopic Göppert-Mayer units [1GM = 10^–50^ cm^4^s/photon]^50^) is based on the following formula^51^:
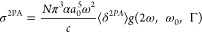
1where *N* is an integer, α is the fine-structure constant, *a*_0_ is the Bohr radius, ω is the photon energy derived from excitation energy ω_0_/2 for two photons of equal energy, *c* is the speed of light, ⟨δ^2PA^⟩ is rotationally averaged two-photon transition moment, *g*(2ω, ω_0_, Γ) is the line shape function, and Γ is the lifetime broadening.

For the particular case of parallel linearly polarized light, the rotationally averaged two-photon transition strengths for the |0⟩ → |*f*⟩ transition, in Hermitian theories, e.g. TD-DFT,^52^ are given by
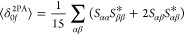
2where *S*_αβ_ are Cartesian components (α, β = *x*, *y*, *z*) of the 2PA transition moment.

In non-Hermitian description, such as RI-CC2 approach, δ^2PA^ for the |0⟩ → |*f*⟩ transition is defined as^48,53^
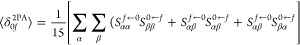
3*S*_αβ_^0 ← *f*^ and *S*_βα_^*f* ← 0^ denote the αβ-th components of the left and right second-order the transition moments, respectively, and are given by
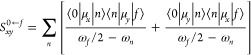
4
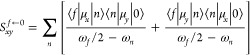
5where μ_*x*_ and μ_*y*_ are Cartesian components of dipole moment operators. The summation runs over all electronic states with ω_*n*_ and ω_*f*_ denoting excitation energies for the intermediate |*n*⟩ and final |*f*⟩ states, respectively. ω_*f*_/2 corresponds to the photon energy ω in the degenerate case. It is worth noting that for Hermitian theories, left and right components of two-photon transition moments are identical.

2PA cross sections for the Lorentzian line shape can be determined as
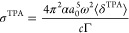
6In this paper, the integer values of Γ and *N* parameters were set as 0.1 eV and 4, respectively.^54^ ⟨δ^TPA^⟩ is readily calculated by Turbomole 7.6 and Dalton 2020 packages.

## Discussion

### De/Methylation Impact on Two-Photon Transition Strengths in the Lowest Excited State at the CC2 Level of Theory

**Figure 2:**
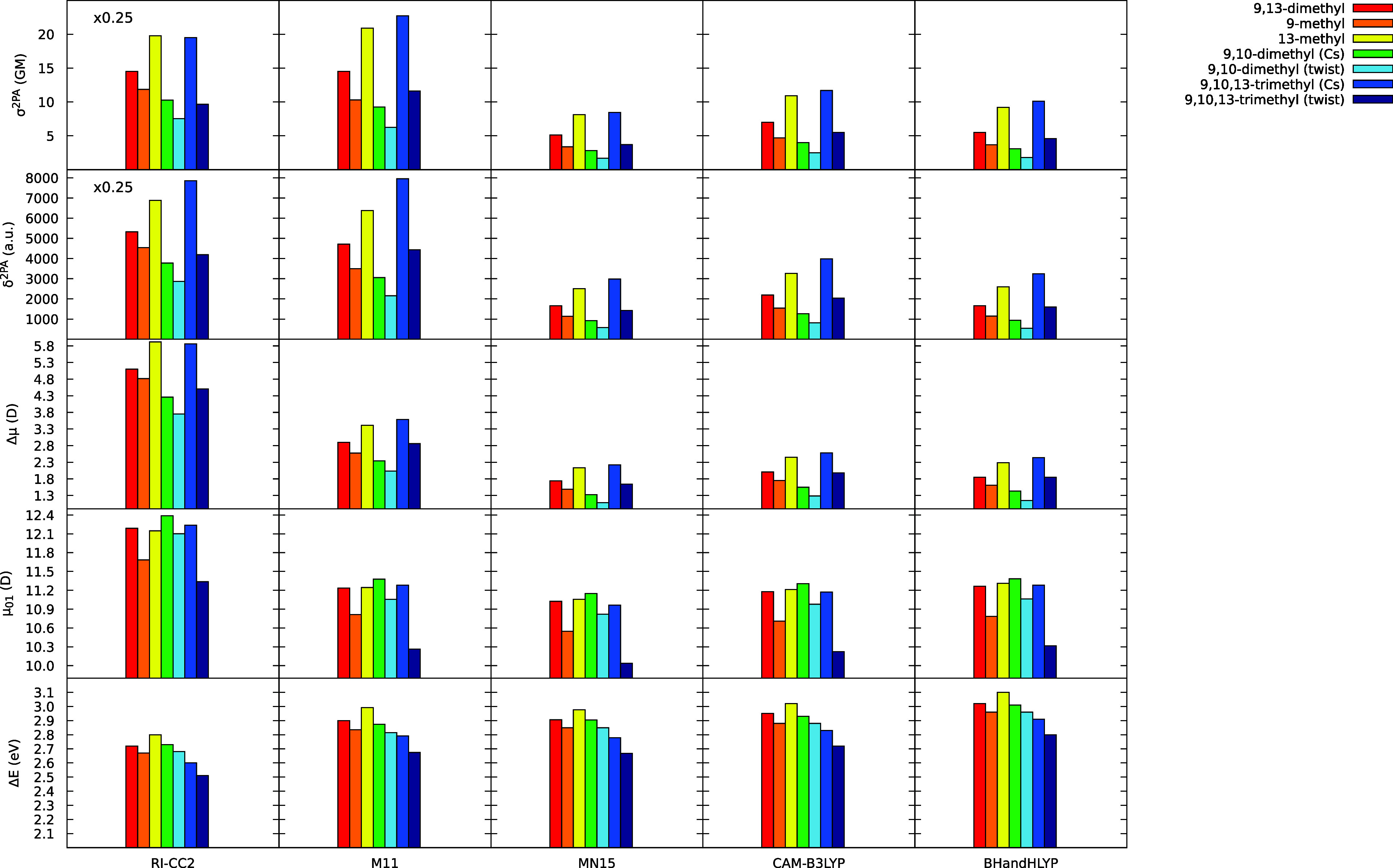
Distribution of 2PA cross sections (σ^2PA^ in GM), two-photon transition strengths (δ^2PA^ in au), change in the permanent dipole moment upon the S0–S1 transition (Δμ in D), S0–S1 transition dipole moment (μ_01_ in D), and 1PA energies (Δ*E* in eV) for RPSB5 models. Methods include RI-CC2/aug-cc-pVDZ and TD-DFT/aug-cc-pVDZ with the M11, MN15, CAM-B3LYP, and BHandHLYP functionals. RI-CC2 δ^2PA^ and σ^2PA^ values were scaled down by 0.25 to facilitate a comparison with the corresponding TD-DFT results.

1PA vertical transition energies, microscopic two-photon transition strengths (δ^2PA^) and macroscopic 2PA cross sections (σ^2PA^), as well as dipole moment properties calculated with the RI-CC2 method, are compiled in Table 1. Since σ^2PA^ response to de/methylation parallels that of δ^2PA^ (see Figures 2 and S1 in the Supporting Information), we focus here on the description of the latter. In all molecular structures, the lowest singlet S1 state is of π → π* nature, dominated by a single reference wave function with double excitation contributions below 12.06%. The influence of the de/methylation in the polyene chain of the rhodopsin chromophore on the single-photon absorption properties has been thoroughly studied by one of us,^22^ hence the discussion in this work will focus on describing the impact of these structural changes on the two-photon properties of RPSB5.

**Table 1:** 1PA Energies (Δ*E*), Two-Photon Transition Strengths (δ^2PA^), 2PA Cross Sections (σ^2PA^), S0–S1 Transition Dipole Moments (μ_01_), the First Excited State (μ_11_) and the Ground State (μ_00_) Permanent Electric Dipole Moments, and Their Differences (Δμ) for RPSB5 Models Calculated Using RI-CC2/aug-cc-pVDZ and TD-DFT/aug-cc-pVDZ Methods with Four Different Functionals^a^

structure	Δ*E* [eV]	δ^2^^PA^ [au]	σ^2^^PA^ [GM]	μ_01_[D]	Δμ [D]	μ_00_ [D]	μ_11_ [D]
RI-CC2							
9,13-dimethyl	2.720	21307	58.0	12.188	5.100	7.837	2.796
9-methyl	2.670	18170	47.4	11.684	4.818	8.102	3.663
13-methyl	2.800	27544	79.1	12.148	5.922	7.044	1.207
9,10-dimethyl (Cs)	2.727	15097	41.1	12.388	4.261	8.312	4.188
9,10-dimethyl (twist)	2.680	11451	30.1	12.102	3.752	7.991	4.424
9,10,13-trimethyl (Cs)	2.600	31434	78.0	12.238	5.865	8.527	2.677
9,10,13-trimethyl (twist)	2.510	16752	38.5	11.337	4.506	7.625	3.258
M11							
9,13-dimethyl	2.914	4713	14.8	11.253	2.899	6.942	4.053
9-methyl	2.851	3494	10.5	10.836	2.576	7.081	4.641
13-methyl	3.005	6378	21.2	11.263	3.407	6.336	2.939
9,10-dimethyl (Cs)	2.888	3055	9.4	11.398	2.344	7.357	5.070
9,10-dimethyl (twist)	2.830	2156	6.4	11.077	2.030	6.991	5.045
9,10,13-trimethyl (Cs)	2.805	7959	23.0	11.301	3.583	7.804	4.231
9,10,13-trimethyl (twist)	2.689	4432	11.8	10.287	2.860	7.008	4.192
MAE	0.182	15653	39.3	0.953	2.075	0.846	1.137
MN15							
9,13-dimethyl	2.918	1655	5.2	11.035	1.739	6.497	4.771
9-methyl	2.861	1136	3.4	10.560	1.492	6.690	5.289
13-methyl	2.988	2504	8.2	11.068	2.133	5.771	3.651
9,10-dimethyl (Cs)	2.916	920	2.9	11.160	1.327	6.993	5.728
9,10-dimethyl (twist)	2.861	573	1.7	10.835	1.088	6.663	5.639
9,10,13-trimethyl (Cs)	2.790	2986	8.6	10.974	2.224	7.259	5.034
9,10,13-trimethyl (twist)	2.683	1423	3.8	10.065	1.642	6.483	4.899
MAE	0.187	18651	48.3	1.198	3.226	1.298	1.828
CAM-B3LYP							
9,13-dimethyl	2.950	2187	7.0	11.179	2.007	6.533	4.537
9-methyl	2.880	1541	4.7	10.710	1.749	6.704	4.975
13-methyl	3.020	3267	10.9	11.212	2.449	5.857	3.417
9,10-dimethyl (Cs)	2.930	1266	4.0	11.307	1.549	7.000	5.451
9,10-dimethyl (twist)	2.880	810	2.5	10.979	1.285	6.652	5.370
9,10,13-trimethyl (Cs)	2.830	3987	11.7	11.173	2.580	7.332	4.753
9,10,13-trimethyl (twist)	2.720	2037	5.5	10.222	1.977	6.567	4.599
MAE	0.215	18094	46.6	1.043	2.947	1.256	1.556
BHandHLYP							
9,13-dimethyl	3.020	1660	5.5	11.263	1.849	6.334	4.495
9-methyl	2.960	1143	3.7	10.785	1.612	6.500	4.908
13-methyl	3.100	2597	9.2	11.313	2.284	5.606	3.331
9,10-dimethyl (Cs)	3.010	939	3.1	11.384	1.430	6.812	5.383
9,10-dimethyl (twist)	2.960	547	1.8	11.063	1.147	6.460	5.315
9,10,13-trimethyl (Cs)	2.910	3239	10.1	11.283	2.435	7.115	4.680
9,10,13-trimethyl (twist)	2.800	1595	4.6	10.315	1.848	6.360	4.524
MAE	0.293	18576	47.8	0.954	3.089	1.464	1.489

a Please note that 1PA energies (Δ*E*) correspond to twice 2PA energies, assuming the latter ones are degenerate.

In comparing all 6 analogues to the native 9,13-dimethyl, profound changes in the δ^2PA^ can be easily observed purely on the basis of the changes in the methylation pattern of the polyene chain. The highest δ^2PA^ value is shown by all planar analogues containing the C-13 methyl group. It is worth noting that the 13-methyl and 9,10,13-trimethyl analogues have higher δ^2PA^ values than the native chromophore by about 30 and 50%, respectively. The addition of the C-9 methyl group seems to have a strong reductive effect on δ^2PA^ as seen in 9,13-dimethyl. A moderate decrease in δ^2PA^ by ca. 3000 au is observed upon the addition of the C-10 methyl group to 9-methyl compound forming 9,10-dimethyl (Cs). In addition, the deviation from planarity leads to a dramatic decrease of the δ^2PA^ by up to over 40% in two investigated analogues 9,10-dimethyl and 9,10,13-trimethyl.

Gaining an insight into macroscopic σ^2PA^ requires knowledge of the quantities Δ*E* and δ^2PA^, where the latter depends on the permanent dipole moments in the ground and excited states and the transition dipole moments. Therefore, looking to the components that contribute toward the δ^2PA^ (see eqs 4 and 5) may give a better understanding of the de/methylation trend, as was described in earlier studies by our group,^55,56^ as well as by others.^54^

When all of the planar structures are compared to the native 9,13-dimethyl, the pattern points to a proclivity for the methylation at the C-13 position to enhance δ^2PA^ values. With the deletion of the C-9 methyl, resulting in the 13-methyl analogue, an enhancement of Δ*E* and Δμ, when compared to the native can be observed (Table 1 and Figure 2). In spite of having the highest Δ*E* at 2.80 eV, 13-methyl still exhibits the second highest δ^2PA^ at 27,544 au, an increase by approximately 6000 au from the native model. The other analogue with methylation at the C-13 position: 9,10,13-trimethyl (Cs) displays the second highest Δμ at 5.865 D, only 0.06 D lower than 13-methyl’s. This, along with the lowest Δ*E* of 2.60 eV, and the second highest μ_01_ of 12.238 D, after 9,10-dimethyl (Cs), places the 9,10,13-trimethyl (Cs) as the brightest δ^2PA^ structure within this study, at 31,434 au Therefore, for the case of analogue 9,10,13-trimethyl (Cs), a combination of higher μ_01_ (by 0.09 D), slightly lower Δμ (by 0.06 D), and much lower ΔE contributed to the higher δ^2PA^ than 13-methyl.

On the opposite end, the 9-methyl analogue exhibits attenuation of Δ*E*, μ_01_, and Δμ properties when compared to 9,13-dimethyl. Analyzing Table 1, one can see that μ_01_ values of all planar structures but one lie in a very narrow range between 12.148–12.388 D, while 9-methyl reveals considerably lower value −11.684 D. In fact, the 9-methyl analogue, despite having a lower Δ*E*, has a much lower δ^2PA^ than the native model, due to having the weakest μ_01_ and the second weakest Δμ of all planar structures. At the same time, 9,10-dimethyl (Cs) exhibits the highest μ_01_, and the second highest Δ*E*, yet it still demonstrates a significantly darker δ^2PA^ signal of 15,097 au with respect to 21,307 au of 9,13-dimethyl. The main difference lies in the decrease of Δμ, which has shifted by ca. 0.8 D compared to the native model. In fact, large dipole moments are exhibited in all structures for the S0 ground state, which varies from 7.044 to 8.527 D and can be found in Table 1. The analogue with the highest S0 dipole moment belonged to the 9,10,13-trimethyl (Cs) structure while the lowest was found in 13-methyl. Upon excitation to the S1 state, the dipole moments reduce significantly to approximately 1.21–4.42 D, yielding an extensive Δμ, signifying a large charge-transfer process. The two smallest S1 dipole moments belonged to 13-methyl at 1.207 D, and 9,10,13-trimethyl at 2.677 D, which resulted in both structures also having the largest Δμ. The significance of Δμ can be exhibited in 9,10-dimethyl (Cs). 9,10-dimethyl (Cs) has slightly higher μ_01_ (0.24 D difference) but lower Δ*E* (0.073 eV) to the corresponding values in 13-methyl, yet it has a much weaker δ^2PA^ signal by over 12,000 au. This is explained by a very large difference in Δμ, where 13-methyl’s is larger by over 1.66 D.

On the other hand, the two lowest δ^2PA^ strengths belong to the two twisted structures of 9,10,13-trimethyl and 9,10-dimethyl. In the 2006 study by Sugihara et al.,^57^ the team observed that the control of absorbance wavelengths is dependent on both the torsion of double bonds, where a 20° twist along the C11=C12 constitutes a redshift of approximately 10 nm and methyl substitution also constitutes a 5–10 nm redshift depending on the position. Additionally, the 2015 study by Walczak et al.^22^ suggests that this torsional angle is also proportional to the magnitude of Δμ. Furthermore, the degree of dihedral symmetry seems to also proportionally affect the δ^2PA^ intensities. When introducing a 10° twist, and thus going from the planar 9,10-dimethyl to a twisted one, a reduction of δ^2PA^ of over 3600 au can be seen, resulting in a nearly 25% decrease, which may be mainly attributed to ca. 12% downshift in Δμ. The reduction in Δμ is due to the decrease in the ground state permanent electric dipole moment (μ_00_) coupled with the increase in the excited state permanent electric dipole moment (μ_11_). Looking over to μ_01_, a more subtle reduction is observed at 0.286 D. The same story is also observed for the 9,10,13-trimethyl structure, where a reduction of over 14,700 au, or the equivalent of approximately 50% is observed. This is, as expected, accompanied by an even larger reduction in all other electronic properties than in 9,10-dimethyl (twist), which includes, Δ*E* by 0.09 eV, Δμ by 1.36 D, and μ_01_ by 0.90 D. However, when comparing only the twisted structures to one another the patterns observed in Walczak et al.^22^ are quite pronounced. The 9,10-dimethyl (twist) analogue with a small twist around C11=C12 bond of 10° has a much lower change in the dipole moment of 3.752 D when compared to the heavier twisted structure (27°) of 9,10,13-trimethyl (twist) with Δμ of 4.506 D. This in turn, results in over 5000 au increase in δ^2PA^ intensities, or approximately 45% increase. In general, the inclusion of the C13-methyl group seems to heavily decrease μ_11_ and in turn increases Δμ and δ^2PA^.

### TD-DFT Performance

In our previous systematic study of reduced size retinal models (RPSB3 and RPSB4), the CAM-B3LYP and BHandHLYP functionals were found to recover the best δ^2PA^ as compared to the reference CC3 out of all functionals investigated, despite its order of magnitude error.^32^ The same DFT functionals were proven to beat the competition while investigating 2PA effects in fluorescent proteins’ chromophores as compared to CC2 reference data.^55^ However, meta-GGA or meta-NGA functionals have not been assessed for these biological chromophores in the context of 2PA. Recently, Grotjahn and Furche,^34^ and independently, Ahmadzadeh et al.^35^ demonstrated that meta-GGA functionals, especially MN15, outperformed range-separated functionals in recovering CC2-based δ^2PA^ and excited state dipole moments for 48 push–pull π-conjugated molecules. A broad palette of various DFT functionals was investigated in search for top-performing functional in a series of coumarin dyes by Elayan et al.^36^ It was shown that range-separated hybrid GGA and hybrid meta-GGA functionals with M11 at the top of the list are superior in predicting δ^2PA^ and σ^2PA^. Even though M11 is worse than BHandHLYP and CAM-B3LYP in recovering RI-CC2 excitation energies, it emerges as more consistent and robust in dipole moments calculations.^36^

In this study, we assessed the four above-mentioned functionals comprising four different functional subclasses: global hybrid GGA (BHandHLYP), long-range-corrected hybrid GGA (CAM-B3LYP), global hybrid meta-NGA (MN15), and range-separated hybrid meta-GGA (M11) with respect to the RI-CC2 results. Hybrid meta-GGA and meta-NGA functionals have never been used in the study of 2PA properties of RPSB compounds. The question is whether these classes of functionals offer any improvement over BHandHLYP and CAM-B3LYP in quadratic-response calculations’ for RPSB5. It is evident from Figures 2 and S1, in the Supporting Information, that all functionals predict the correct trends of increasing δ^2PA^, by ca. 30% at the CC2 and up to 50% at TD-DFT, upon the demethylation at the C9 position, and decreasing δ^2PA^, by ca. 15% at CC2 and up to over 30% at TD-DFT, upon the analogous demethylation at the C13 position. Moreover, a rather dramatic effect of δ^2PA^ quenching due to the replacement of the C13-methyl with the C10-methyl group, leading to 9,10-dimethyl (Cs), is also captured by TD-DFT, although, as above, the change is clearly exaggerated (30% at CC2 vs over 40% at TD-DFT).

The δ^2PA^ values of the twisted analogues are downshifted when compared to any of the planar chromophores, except 9,10-dimethyl (Cs), at the CC2, but this trend is not recovered by TD-DFT. In particular, all functionals correctly predict 9,10-dimetyl (twist) to have the lowest δ^2PA^ among the studied compounds but wrongly place δ^2PA^ of 9,10,13-trimethyl (twist), regardless of the functional used, above the corresponding values for 9-methyl and 9,10-dimethyl (Cs) and close to 9,13-dimethyl. As revealed by Figure 2, CAM-B3LYP, BHandHLYP, and MN15 functionals strongly exaggerate the relative difference between the native and the brightest (9,10,13-trimethyl (Cs)) and the darkest (9,10-dimethyl (twist)) chromophore in terms of δ^2PA^. M11 does perform better, but still the curve displaying the impact of de/methylation pattern on δ^2PA^ is too steep when compared to the corresponding CC2 one (see Figure S1 in the Supporting Information).

The absolute values of δ^2PA^ calculated by TD-DFT using various functionals reveal that M11 is the best-performing functional underestimating the CC2 values by a factor of 3.8–5.3 instead of a factor of 8.2–14.1 as in the case of CAM-B3LYP, and 9.7–20.9 for BHandHLYP and MN15 functionals, with the latter one being the worst among all functionals. Contrary to what can be seen in studies by Ahmazadeh et al.^35^ and Grotjahn and Furche^34^ for a set of organic molecules, MN15 does not confirm that it is superior to CAM-B3LYP in 2PA calculations of retinals. Instead, our top-performing functional—M11 was recommended earlier by Brown and co-workers for coumarin dyes.^36^

TD-DFT does well to capture the excitation energy trend predicted by CC2 with the notable exception of 9,13-dimethyl and 9,10-dimethyl (Cs) order. At the CC2 level of theory, 9,10-dimethyl (Cs) has a bit higher excitation energy (by 0.007 eV) while functionals predict the opposite, albeit by a small margin 0.01–0.02 eV. The excitation energies produced by CAM-B3LYP and BHandHLYP are within 0.2–0.3 eV from the corresponding CC2 values. This error is reduced in meta-GGA/meta-NGA functionals by ca. 0.1 eV.

Jacquemin considered a set of over 30 medium and large molecules to assess the quality of the dipole moments obtained with 16 different exchange-correlation functionals.^58^ He found that on average, DFT underestimates ground state dipole moments and TD-DFT mostly overestimates excited state dipole moments with larger computed magnitudes with respect to CC2 results. Moreover, the impact of the selected functional becomes considerable only for charge-transfer states. This pattern holds here with μ_00_ showing 0.6–1.6 D downshift and μ_11_ upshift by even 2.4 D. As a result, the magnitudes of Δμ are significantly underestimated and their MAEs increase in the order: M11 (MAE = 2.075 D) < CAM-B3LYP (MAE = 2.947 D) < BHandHLYP (MAE = 3.089 D) < MN15 (MAE = 3.226 D).

The pattern of a very narrow range of transition dipole moments (0.29 D) shared by five RPSB5 compounds with two outliers, 9-methyl and 9,10,13-trimethyl (twist), displaying significantly lower values, by ca. 0.50 and 0.85 D, respectively, than their 9,13-dimethyl counterpart, is reproduced qualitatively by all functionals; however, they are plagued with ca. 1 D error in case of M11, CAM-B3LYP, and BHandHLYP. Even larger underestimation, ca. 1.2 D, is observed for MN15.

Judging by the results described above, it seems that the best performance of M11 in predicting 2PA strengths and cross sections stems mainly from the much higher quality of permanent dipole moments, both in the ground and the lowest excited state.

It is worth noting that the two-state model, in which δ^2PA^ is proportional to the square of μ_01_ and Δμ,^59^ works very well in capturing the impact of de/methylation in the RPSB5 backbone at both CC2 and TD-DFT levels of theory with an almost perfect correlation between δ^2PA^ calculated using quadratic response theory and two-state model (see Figure S4 in the Supporting Information).

**Figure 3:**
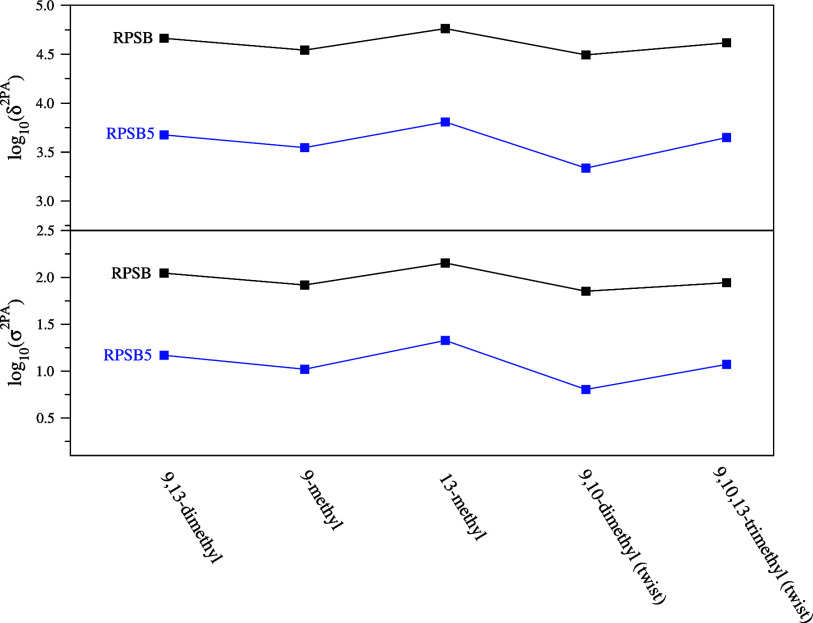
Impact of de/methylation on δ^2PA^ (in au; top panel) and σ^2PA^ (in GM; bottom panel) calculated for the lowest excited state in RPSB and RPSB5 models. σ^2PA^ and δ^2PA^ are represented by their respective log_10_ to allow for direct comparison.

One may wonder whether the presence of the β-ionone ring in the polyene chain (RPSB; see Figure 1) will change the effect of de/methylation on the 2PA properties observed in the RPSB5 models. To verify this, we performed TD-DFT/M11/aug-cc-pVDZ calculations for five RPSBs (for results see Figure S5 and Table S10 in the Supporting Information). The results showed that even though, the absolute δ^2PA^ values are 1 order of magnitude larger than in the RPSB5 models, mainly due to more than twice the change value of the dipole moments after excitation and dramatically reduced excitation energies (by over 0.4 eV) compared to RPSB5s, which in turn is consequence of the longer conjugated chain (six double CC bonds in RPSB vs five in RPSB5), the relative changes in δ^2PA^ (and also in σ^2PA^) upon de/methylation remain similar as shown in Figure 3.

## 4 Conclusions

We have presented here an investigation onto the impact of de/methylation of polyene chain on 2PA properties for multiple retinal protonated Schiff base (RPSB5) models in the RI-CC2 ansatz. The results of our work displayed that δ^2PA^ follow nearly exactly the trend designated by Δμ, where the de/methylation of the polyene chain enhances the change in dipole moments from the ground to the first excited state for the structures that include the C13 methyl group, namely, 13-methyl and 9,10,13-trimethyl (Cs). Furthermore, the dependence of δ^2PA^ on μ_01_ and Δ*E* can also be clearly observed.

The twisting of the C10–C11 and C11=C12 bonds manifested in both 9,10-dimetyl (twist) and 9,10,13-trimethyl (twist) structures did dramatically suppress the structures’ Δ*E* and Δμ, and to a lower extent μ_01_, when compared to their planar counterparts, resulting in ca. 40% reduction of δ^2PA^.

Among the investigated functionals: M11, MN15, CAM-B3LYP, and BHandHLYP, following the principle of “choosing bad over worse”,^60^ range-separated meta-GGA functional M11 comes undeniably at the top. δ^2PA^ and σ^2PA^ values predicted by M11 are almost three times larger than the corresponding values by other functionals and thus much closer to the reference CC2 values. The reason why M11 outperforms other functionals in predicting 2PA properties of RPSBs in the lowest excited state is its ability to give a much more accurate description of both ground- and excited-state dipole moments.

## Supporting Information Available

Impact of de/methylation on σ^2PA^ and δ^2PA^; RI-CC2 and TD-DFT results for 1PA and 2PA properties for higher (S2–S5) excited states; RI-CC2 diagnostics; σ^2PA^ and δ^2PA^ for the lowest excited state obtained using two-state model; 1PA and 2PA results from TDDFT/M11 calculations for full RPSB models; Cartesian coordinates of RPSB models (PDF)

## References

[ref1] Osero A. R.; Callender R. H. Resonance raman spectroscopy of rhodopsin in retinal disk membranes. Biochemistry 1974, 13 (20), 4243–4248. 10.1021/bi00717a027. 4472288

[ref2] Ovchinnikov Y. A. Bioorganic chemistry of rhodopsins. Pure Appl. Chem. 1986, 58 (5), 725–736. 10.1351/pac198658050725.

[ref3] Wald G. Molecular basis of visual excitation. Science 1968, 162 (3850), 230–239. 10.1126/science.162.3850.230. 4877437

[ref4] Gozem S.; Luk H. L.; Schapiro I.; Olivucci M. Theory and simulation of the ultrafast double-bond isomerization of biological chromophores. Chem. Rev. 2017, 117, 13502 10.1021/acs.chemrev.7b00177. 29083892

[ref5] Walczak E.; Szefczyk B.; Andruniów T. Impacts of retinal polyene (de)methylation on the photoisomerization mechanism and photon energy storage of rhodopsin. Phys. Chem. Chem. Phys. 2015, 17 (26), 17169–17181. 10.1039/C5CP01939G. 26074351

[ref6] List N. H.; Olsen J. M. H.; Kongsted J. Excited states in large molecular systems through polarizable embedding. Phys. Chem. Chem. Phys. 2016, 18 (30), 20234–20250. 10.1039/C6CP03834D. 27416749

[ref7] Kim H. W.; Kim K.; Park S. W.; Rhee Y. M. Quantum chemistry for studying electronic spectroscopy and dynamics of complex molecular systems. In Molecular Spectroscopy: A Quantum Chemistry Approach; Wiley, 2019; Vol 1; pp. 79–118.

[ref8] Manathunga M.; Yang X.; Olivucci M. Electronic state mixing controls the photoreactivity of a rhodopsin with all-trans chromophore analogues. J. Phys. Chem. Lett. 2018, 9 (21), 6350–6355. 10.1021/acs.jpclett.8b02550. 30336038 PMC6261349

[ref9] Yang X.; Manathunga M.; Gozem S.; Léonard J.; Andruniów T.; Olivucci M. Quantum-classical simulations of rhodopsin reveal excited-state population splitting and its e↑ects on quantum eciency. Nat. Chem. 2022, 14, 441–449. 10.1038/s41557-022-00892-6. 35241801 PMC8983576

[ref10] Pepperberg D. R. Generation of rhodopsin and “artificial” visual pigments in electrophysiologically active photoreceptors. Methods Enzymol. 1982, 81, 452–459. 10.1016/s0076-6879(82)81063. 7098892

[ref11] Ganter U. M.; Schmid E. D.; Perez-Sala D.; Rando R. R.; Siebert F. Removal of the 9-methyl group of retinal inhibits signal transduction in the visual process. A fourier transform infrared and biochemical investigation. Biochemistry 1989, 28 (14), 5954–5962. 10.1021/bi00440a036. 2505843

[ref12] Wang Q.; Kochendoerfer G. G.; Schoenlein R. W.; Verdegem P. J. E.; Lugtenburg J.; Mathies R. A.; Shank C. V. Femtosecond spectroscopy of a 13-demethylrhodopsin visual pigment analogue:the role of nonbonded interactions in the isomerization process. J. Phys. Chem. 1996, 100 (43), 17388–17394. 10.1021/jp961150s.

[ref13] Vogel R.; Fan G.-B.; Sheves M.; Siebert F. The molecular origin of the inhibition of transducin activation in rhodopsin lacking the 9-methyl group of the retinal chromophore: A UVVis and FTIR spectroscopic study. Biochemistry 2000, 39 (30), 8895–8908. 10.1021/bi000852b. 10913302

[ref14] Vogel R.; Lüdeke S.; Siebert F.; Sakmar T. P.; Hirshfeld A.; Sheves M. Agonists and partial agonists of rhodopsin: Retinal polyene methylation affects receptor activation. Biochemistry 2006, 45 (6), 1640–1652. 10.1021/bi052196r. 16460011

[ref15] Verdegem P. J. E.; Bovee-Geurts P. H. M.; de Grip W. J.; Lugtenburg J.; de Groot H. J. M. Retinylidene ligand structure in bovine rhodopsin, metarhodopsin-i, and 10-methylrhodopsin from internuclear distance measurements using 13c-labeling and 1-d rotational resonance MAS NMR. Biochemistry 1999, 38 (35), 11316–11324. 10.1021/bi983014e. 10471281

[ref16] Kochendoerfer G. G.; Verdegem P. J. E.; van der Hoef I.; Lugtenburg J.; Mathies R. A. Retinal analog study of the role of steric interactions in the excited state isomerization dynamics of rhodopsin. Biochemistry 1996, 35 (50), 16230–16240. 10.1021/bi961951l. 8973196

[ref17] Gartner W.; Ternieden S. In’uence of a steric hindrance in the chromophore of rhodopsin on the quantum yield of the primary photochemistry. J. Photochem. Photobiol., B 1996, 33 (1), 83–86. 10.1016/1011-1344(95)07225-X.

[ref18] Koch D.; Gartner W. Steric hindrance between chromophore substituents as the driving force of rhodopsin photoisomerization: 10-methyl-13-demethyl retinal containing rhodopsin. Photochem. Photobiol. 1997, 65 (1), 181–186. 10.1111/j.1751-1097.1997.tb01896.x. 9066300

[ref19] DeLange F.; Bovee-Geurts P. H.; Portier J. V. M. D.; Verdegem P. J.; Lugtenburg J.; de Grip W. J. An additional methyl group at the 10-position of retinal dramatically slows down the kinetics of the rhodopsin photocascade. Biochemistry 1998, 37 (5), 1411–1420. 10.1021/bi972397y. 9477970

[ref20] Vico L. D.; Page C. S.; Garavelli M.; Bernardi F.; Basosi R.; Olivucci M. Reaction path analysis of the “tunable” photoisomerization selectivity of free and locked retinal chromophores. J. Am. Chem. Soc. 2002, 124 (15), 4124–4134. 10.1021/ja017502c. 11942852

[ref21] Valsson O.; Angeli C.; Filippi C. Excitation energies of retinal chromophores: Critical role of the structural model. Phys. Chem. Chem. Phys. 2012, 14, 11015–11020. 10.1039/c2cp41387f. 22782521

[ref22] Walczak E.; Szefczyk B.; Andruniów T. Geometries and vertical excitation energies in retinal analogues resolved at the CASPT2 level of theory: Critical assessment of the performance of CASSCF, CC2, and DFT methods. J. Chem. Theory Comput. 2013, 9 (11), 4915–4927. 10.1021/ct400423u. 26583410

[ref23] Send R.; Sundholm D. Coupled-cluster studies of the lowest excited states of the 11-cis-retinal chromophore. Phys. Chem. Chem. Phys. 2007, 9, 2862–2867. 10.1039/b616137e. 17538731

[ref24] Tuna D.; Lefrancois D.; Wolański S.; Gozem I.; Schapiro T.; Andruniów A.; Dreuw M. O. Assessment of approximate coupled-cluster and algebraic-diagrammatic-construction methods for ground- and excited-state reaction paths and the conical-intersection seam of a retinal-chromophore model. J. Chem. Theory Comput. 2015, 11, 5758 10.1021/acs.jctc.5b00022. 26642989

[ref25] Aquino A. J. A.; Barbatti M.; Lischka H. Excited-state properties and environmental effects for protonated Schiff bases: A theoretical study. ChemPhysChem 2006, 7 (10), 2089–2096. 10.1002/cphc.200600199. 16941558

[ref26] de Grip W. J.; Ganapathy S. Rhodopsins: An excitingly versatile protein species for research, development and creative engineering. Front. Chem. 2022, 10, 2296–2646. 10.3389/fchem.2022.879609. PMC9257189 35815212

[ref27] Nakano M.; Kooriya T.; Kuragaito T.; Egami C.; Kawata Y.; Tsuchimori M.; Watanabe O. Three-dimensional patterned media for ultrahigh-density optical memory. Appl. Phys. Lett. 2004, 85 (2), 176–178. 10.1063/1.1771800.

[ref28] Kulkarni R. U.; Vandenberghe M.; Thunemann M.; James F.; Andreassen O. A.; Djurovic S.; Devor A.; Miller E. W. In vivo two-photon voltage imaging with sulfonated rhodamine dyes. ACS Cent. Sci. 2018, 4 (10), 1371–1378. 10.1021/acscentsci.8b00422. 30410975 PMC6202643

[ref29] Palczewska G.; Boguslawski J.; Stremplewski P.; Kornaszewski L.; Zhang J.; Dong Z.; Liang X.-X.; Gratton E.; Vogel A.; Wojtkowski M.; Palczewski K. Noninvasive two-photon optical biopsy of retinal fluorophores. Proc. Natl. Acad. Sci. U.S.A. 2020, 117 (3), 22532–22543. 10.1073/pnas.2007527117. 32848058 PMC7486747

[ref30] An J. M.; Kim S. H.; Kim D. Recent advances in two-photon absorbing probes based on a functionalized dipolar naphthalene platform. Org. Biomol. Chem. 2020, 18 (23), 4288–4297. 10.1039/D0OB00515K. 32242192

[ref31] Sneskov K.; Olsen J. M. H.; Schwabe T.; Hättig C.; Christiansen O.; Kongsted J. Computational screening of one- and two-photon spectrally tuned channelrhodopsin mutants. Phys. Chem. Chem. Phys. 2013, 15 (20), 7567 10.1039/c3cp44350g. 23588588

[ref32] Sirimatayanant S.; Andruniów T. Benchmarking two-photon absorption strengths of rhodopsin chromophore models with CC3 and CCSD methodologies: An assessment of popular density functional approximations. J. Chem. Phys. 2023, 158 (9), 094106 10.1063/5.0135594. 36889953

[ref33] Weigend F.; Köhn A.; Hättig C. Efficient use of the correlation consistent basis sets in resolution of the identity MP2 calculations. J. Chem. Phys. 2002, 116 (8), 3175–3183. 10.1063/1.1445115.

[ref34] Grotjahn R.; Furche F. Gauge-invariant excited-state linear and quadratic response properties within the meta-generalized gradient approximation. J. Chem. Theory Comput. 2023, 19 (15), 4897–4911. 10.1021/acs.jctc.3c00259. 37399786

[ref35] Ahmadzadeh K.; Li X.; Rinkevicius Z.; Norman P.; Zaleśny R. Toward accurate two-photon absorption spectrum simulations: Exploring the landscape beyond the generalized gradient approximation. J. Phys. Chem. Lett. 2024, 15 (4), 969–974. 10.1021/acs.jpclett.3c03513. 38252270 PMC10839899

[ref36] Elayan I. A.; Rib L.; Mendes R. A.; Brown A. Beyond explored functionals: A computational journey of two-photon absorption. ChemRxiv, 2024. 10.26434/chemrxiv-2024-gm4g2. 38648613

[ref37] Christiansen O.; Koch H.; Jo̷rgensen P. The second-order approximate coupled cluster singles and doubles model CC2. Chem. Phys. Lett. 1995, 243 (5–6), 409–418. 10.1016/0009-2614(95)00841-Q.

[ref38] Hättig C.; Weigend F. CC2 excitation energy calculations on large molecules using the resolution of the identity approximation. J. Chem. Phys. 2000, 113 (13), 5154–5161. 10.1063/1.1290013.

[ref39] Becke A. D. New mixing of hartree-fock and local density-functional theories. J. Chem. Phys. 1993, 98 (2), 1372–1377. 10.1063/1.464304.

[ref40] Yanai T.; Tew D. P.; Handy N. C. A new hybrid exchange-correlation functional using the coulomb-attenuating method (CAM-B3LYP). Chem. Phys. Lett. 2004, 393 (1–3), 51–57. 10.1016/j.cplett.2004.06.011.

[ref41] Yu H. S.; He X.; Li S. L.; Truhlar D. MN15: a kohn-sham global-hybrid exchange-correlation density functional with broad accurcy for multi-reference and single-reference systems and noncovalent interactions. Chem. Sci. 2016, 7 (8), 5032–5051. 10.1039/C6SC00705H. 30155154 PMC6018516

[ref42] Peverati R.; Truhlar D. G. Improving the accuracy of hybrid meta-GGA density functionals by range separation. J. Phys. Chem. Lett. 2011, 2 (21), 2810–2817. 10.1021/jz201170d.

[ref43] Lehtola S.; Steigemann C.; Oliveira M. J. T.; Marques M. A. L. Recent developments in libxc - a comprehensive library of functionals for density functional theory. Software X 2018, 7, 1–5. 10.1016/j.softx.2017.11.002.

[ref44] Balasubramani S. G.; Chen G. P.; Coriani S.; Diedenhofen M.; Frank M. S.; Franzke Y. J.; Furche F.; Grotjahn R.; Harding M. E.; Hättig C.; Hellweg A.; Helmich-Paris B.; Holzer C.; Huniar U.; Kaupp M.; Khah A. M.; Khani S. K.; Müller T.; Mack F.; Nguyen B. D.; Parker S. M.; Perlt E.; Rappoport D.; Reiter K.; Roy S.; Rückert M.; Schmitz G.; Sierka M.; Tapavicza E.; Tew D. P.; van Wüllen C.; Voora V. K.; Weigend F.; Wodyński A.; T J. M. Y. Turbomole: Modular program suite for ab initio quantum-chemical and condensed-matter simulations. J. Chem. Phys. 2020, 152 (18), 184107 10.1063/5.0004635. 32414256 PMC7228783

[ref45] TURBOMOLE V7.5 2020, a development of University of Karlsruhe and Forschungszentrum Karlsruhe GmbH, 1989–2007; TURBOMOLE GmbH, since 2007; available from https://www.turbomole.org, 2020.

[ref46] Aidas K.; Angeli C.; Bak K. L.; Bakken V.; Bast R.; Boman L.; Christiansen O.; Cimiraglia R.; Coriani S.; Dahle P.; et al. The Dalton Quantum Chemistry Program System. Wiley Interdiscip. Rev. Comput. Mol. Sci. 2014, 4 (3), 269–284. 10.1002/wcms.1172. 25309629 PMC4171759

[ref47] Dalton, A Molecular Electronic Structure Program (Release v2020.0, 2020, see http://daltonprogram.org.

[ref48] Friese D. H.; Hättig C.; Ruud K. Calculation of two-photon absorption strengths with the approximate coupled cluster singles and doubles model CC2 using the resolution-of-identity approximation. Phys. Chem. Chem. Phys. 2012, 14 (3), 1175–1184. 10.1039/C1CP23045J. 22130199

[ref49] Hättig C.; Köhn A. Transition moments and excited-state ↓rst-order properties in the coupled-cluster model CC2 using the resolution-of-the-identity approximation. J. Chem. Phys. 2002, 117 (15), 6939–6951. 10.1063/1.1506918.

[ref50] Göppert-Mayer M. Über Elementarakte mit zwei Quantensprüngen. Ann. Phys. 1931, 9, 273–295. 10.1002/andp.19314010303.

[ref51] Peticolas W. L. Multiphoton Spectroscopy. Annu. Rev. Phys. Chem. 1967, 18, 233–260. 10.1146/annurev.pc.18.100167.001313.

[ref52] Sałek P.; Vahtras O.; Gus J.; Luo Y.; Helgaker T.; Ågren H. Calculations of Two-Photon Absorption Cross Sections by Means of Density-Functional Theory. Chem. Phys. Lett. 2003, 374, 446–452. 10.1016/S0009-2614(03)00681-X.

[ref53] Hättig C.; Christiansen O.; Jorgensen P. Multiphoton Transition Moments and Absorption Cross Sections in Coupled Cluster Response Theory Employing Variational Transition Moments Functionals. J. Chem. Phys. 1998, 108, 8331–8354. 10.1063/1.476261.

[ref54] Beerepoot M. T. P.; Friese D. H.; List N. H.; Kongsted J.; Ruud K. Benchmarking two-photon absorption cross sections: Performance of CC2 and CAM-B3LYP. Phys. Chem. Chem. Phys. 2015, 17 (29), 19306–19314. 10.1039/C5CP03241E. 26139162

[ref55] Grabarek D.; Andruniów T. Assessment of functionals for TDDFT calculations of one- and two-photon absorption properties of neutral and anionic fluorescent proteins chromophores. J. Chem. Theory Comput. 2019, 15 (1), 490–508. 10.1021/acs.jctc.8b00769. 30485096

[ref56] Grabarek D.; Andruniów T. Illuminating the origins of two-photon absorption properties in fluorescent protein chromophores. Int. J. Quantum Chem. 2020, 120 (3), e26086 10.1002/qua.26086.

[ref57] Sugihara M.; Hufen J.; Buss V. Origin and consequences of steric strain in the rhodopsin binding pocket. Biochemistry 2006, 45 (3), 801–810. 10.1021/bi0515624. 16411756

[ref58] Jacquemin D. Excited-state dipole and quadrupole moments: Td-dft versus cc2. J. Chem. Theory Comput. 2016, 12, 3993–4003. 10.1021/acs.jctc.6b00498. 27385324 PMC4980690

[ref59] Alam M. M.; Chattopadhyaya M.; Chakrabarti S.; Ruud K. Chemical control of channel interference in two-photon absorption process. Acc. Chem. Res. 2014, 47, 1604–1612. 10.1021/ar500083f. 24758397

[ref60] Chołuj M.; Alam M. M.; Beerepoot M. T. T.; Sitkiewicz S. P.; Matito E.; Ruud K.; Zaleśny R. Choosing bad versus worse: Predictions of two-photon-absorption strengths based on popular density functional approximations. J. Chem. Theory Comput. 2022, 18 (2), 1046–1060. 10.1021/acs.jctc.1c01056. 35080389 PMC8830054

