# Supplementary material for: Tuning Two-Photon Absorption in Rhodopsin Chromophore via Backbone Modification: The Story Told by CC2 and TD-DFT
Source: J Chem Theory Comput. 2024 Sep 13;20(18):8118–26. doi: 10.1021/acs.jctc.4c00675 (PMC11428129; doi:10.1021/acs.jctc.4c00675)
Supplement: Supplementary file 1 — ct4c00675_si_001.pdf [file ct4c00675_si_001.pdf]

The Supporting Information for: Tuning Two-Photon Absorption in Rhodopsin Chromophore via Backbone Modification. The Story Told by CC2 and TD-DFT

Saruti Sirimatayanant[a], Tadeusz Andruniów*[a]

[a] *Institute of Advanced Materials, Department of Chemistry, Wroclaw University of Science and Technology, Wyb. Wyspiańskiego 27, Wrocław, 50-370, Wrocław, Poland*

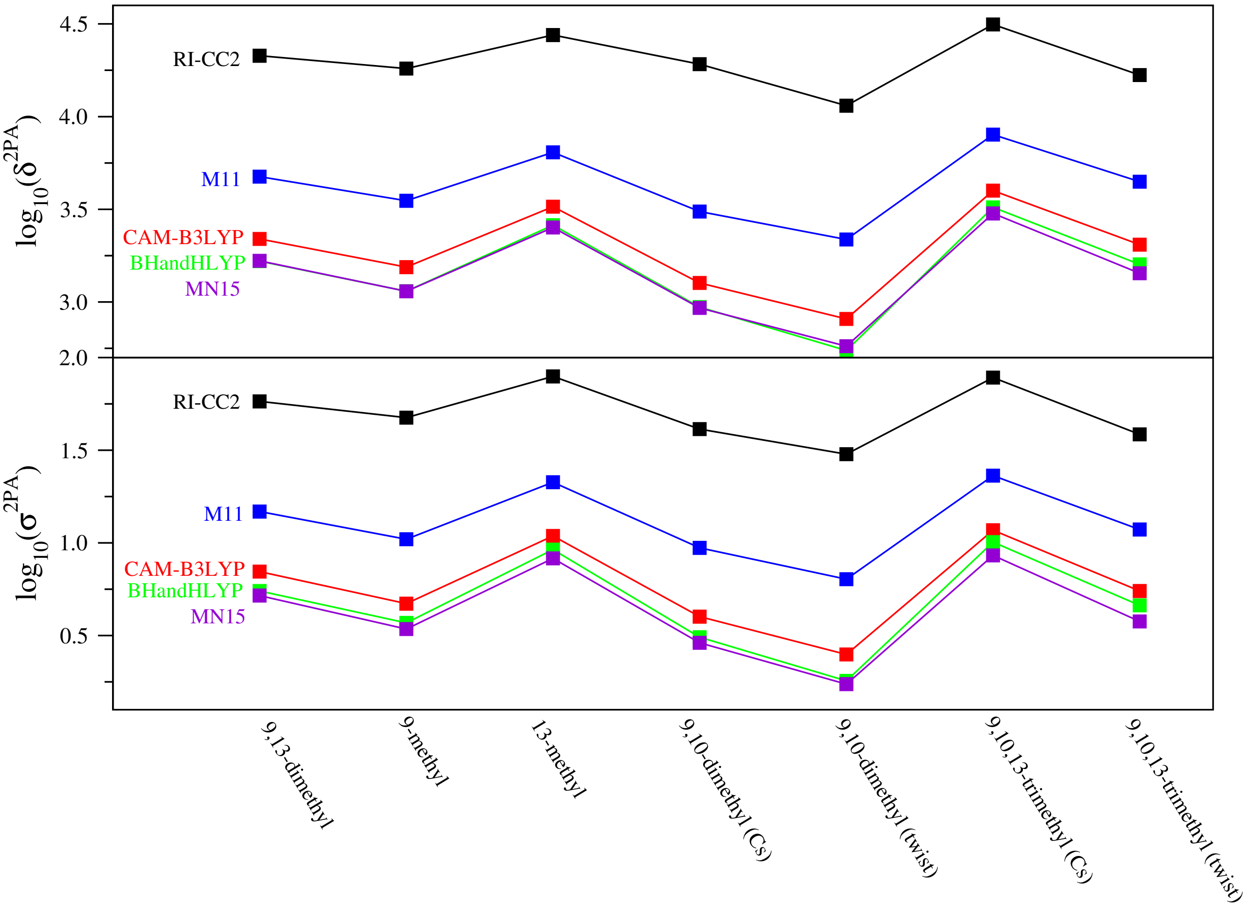

Figure S1: The impact of de/methylation on σ^{2PA} (GM) and δ^{2PA} (a.u.) calculated for the lowest excited state of RPSB5 models. σ^{2PA} and δ^{2PA} are represented by their $\log_{10}$ to allow the direct comparison.

*Corresponding author

Email addresses: saruti.sirimatayanant@pwr.edu.pl (Saruti Sirimatayanant), tadeusz.andruniow@pwr.edu.pl (Tadeusz Andruniów*)

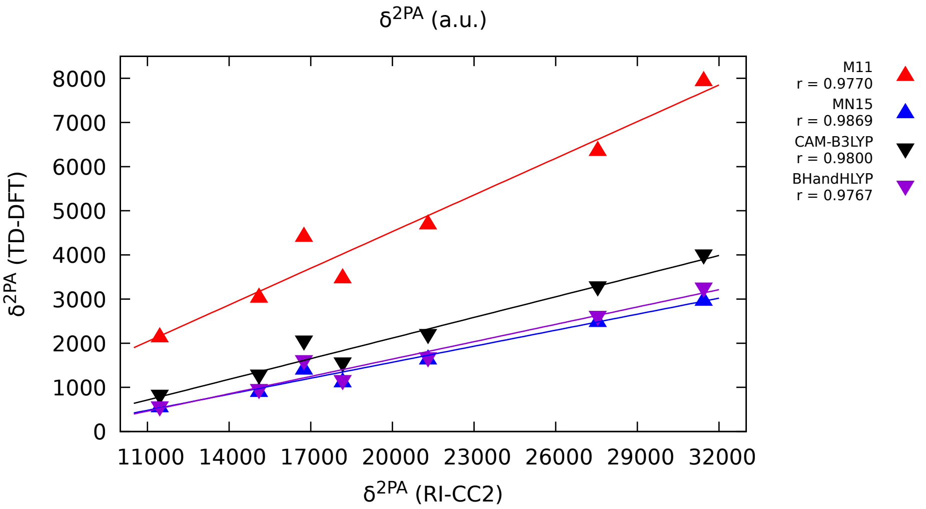

Figure S2: δ^{2PA} of the lowest excited state of all RPSB5 models within this study, calculated at various TD-DFT methods against RI-CC2. Additional statistics corresponding to M11: slope: 0.277 ± 0.027, standard deviation (X and Y): 7070.36 and 2002.85, regression constant: -1006 ± 575.2. MN15: slope: 0.121 ± 0.009, standard deviation (X and Y): 7070.36 and 866.51, regression constant: -849.8 ± 188.1. CAM-B3LYP: slope: 0.156 ± 0.014, standard deviation (X and Y): 7070.36 and 1124.28, regression constant: -999.2 ± 301.3. BHandHLYP: slope: 0.131 ± 0.013, standard deviation (X and Y): 7070.36 and 948.68, regression constant: -979.4 ± 274.3.

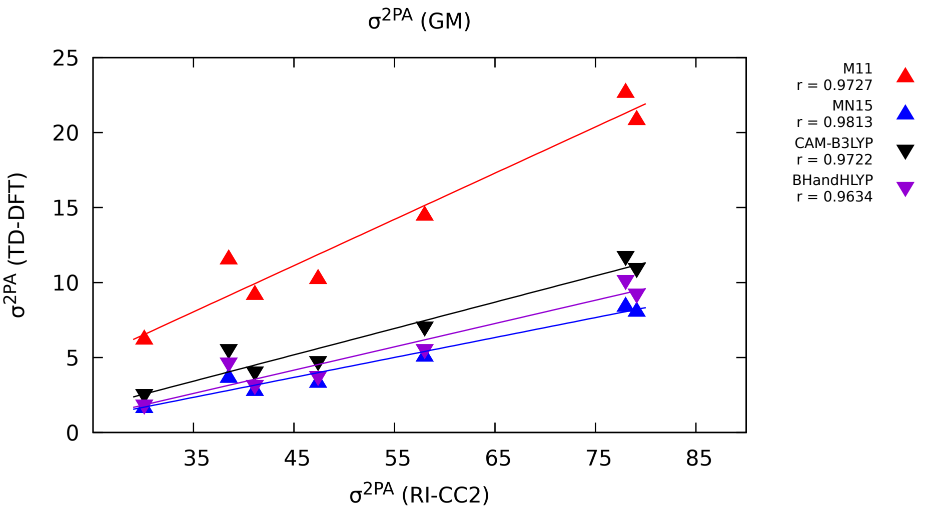

Figure S3: σ^{2PA} of the lowest excited state of all RPSB5 models within this study, calculated at various TD-DFT methods against RI-CC2. Additional statistics corresponding to M11: slope: 0.308 ± 0.033, standard deviation (X and Y): 19.316 and 6.122, regression constant: -2.743 ± 1.844. MN15: slope: 0.133 ± 0.012, standard deviation (X and Y): 19.316 and 2.617, regression constant: -2.309 ± 0.655. CAM-B3LYP: slope: 0.176 ± 0.019, standard deviation (X and Y): 19.316 and 3.489, regression constant: -2.723 ± 1.062. BHandHLYP: slope: 0.156 ± 0.019, standard deviation (X and Y): 19.316 and 3.118, regression constant: -2.839 ± 1.086.

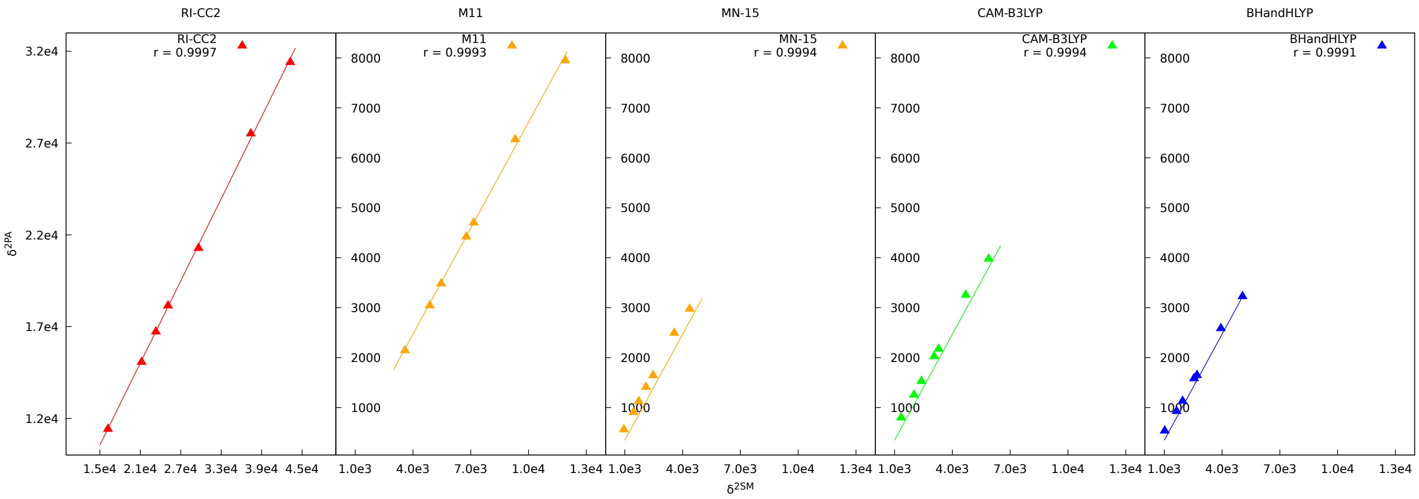

Figure S4: Comparison of two-photon transition strengths from response theory (δ^{2PA}) and two-state model (δ^{2SM}) for RPSB5 models obtained at different levels of theory.

Table S1: TD-DFT results for 2PA properties of the lowest excited state of RPSB5 models calculated using M11 and MN15 functionals with the current-density response enabled for meta-GGA calculations. MAE calculated with respect to the RI-CC2 results.

Structure	M11 δ^{2PA} [a.u.]	M11 σ^{2PA} [GM]	MN15 δ^{2PA} [a.u.]	MN15 σ^{2PA} [GM]
9,13-dimethyl	4745	14.8	1667	5.2
9-methyl	3514	10.5	1142	3.4
13-methyl	6420	21.2	2523	8.2
9,10-dimethyl (Cs)	3077	9.4	928	2.9
9,10-dimethyl (twist)	2172	6.4	577	1.7
9,10,13-trimethyl (Cs)	7997	23.0	3003	8.6
9,10,13-trimethyl (twist)	4457	11.8	1429	3.8
MAE	15625	39.3	18641	48.3

Table S2: Oscillator strengths for the lowest-energy S0-S1 excitation for RPSB5 models.

Structure	RI-CC2	M11	MN15	CAM-B3LYP	BHandHLYP
9,13-dimethyl	1.535	1.399	1.347	1.396	1.455
9-methyl	1.382	1.270	1.210	1.254	1.308
13-methyl	1.567	1.446	1.388	1.441	1.503
9,10-dimethyl (Cs)	1.587	1.423	1.377	1.422	1.481
9,10-dimethyl (twist)	1.488	1.317	1.274	1.315	1.374
9,10,13-trimethyl (Cs)	1.478	1.358	1.274	1.337	1.404
9,10,13-trimethyl (twist)	1.221	1.079	1.031	1.078	1.129

Table S3: RI-CC2 diagnostics - %T1, for the 5 lowest excited states in RPSB5 models.

Structure	S1	S2	S3	S4	S5
9,13-dimethyl	88.37	84.80	85.44	87.83	90.73
9-methyl	88.57	84.51	87.77	86.27	90.95
13-methyl	88.34	85.07	87.24	86.02	85.52
9,10-dimethyl (Cs)	88.37	84.39	85.22	88.00	90.51
9,10-dimethyl (twist)	88.48	84.06	86.60	87.15	88.63
9,10,13-trimethyl (Cs)	87.94	84.90	84.98	88.00	90.61
9,10,13-trimethyl (twist)	88.24	85.22	84.85	88.10	85.26

Table S4: 1PA and 2PA properties for the higher excited states (S2-S5) in RPSB5 models calculated using RI-CC2.

			**RI-CC2**				
Structure	E.S. [no.]	ΔE [eV]	δ^{2PA} [a.u.]	σ^{2PA} [GM]	μ_{01} [D]	$\Delta\mu$ [D]	μ_{11} [D]
9,13-dimethyl	2	4.357	-27059	-	2.652	3.676	4.268
	3	5.046	-	-	3.136	4.603	3.627
	4	5.272	-	-	0.779	1.759	6.080
	5	5.613	9013	104.0	0.103	12.727	4.891
9-methyl	2	4.429	-226076	-	3.428	2.431	5.746
	3	5.042	-	-	3.895	3.017	5.892
	4	5.382	-	-	1.159	5.641	2.941
	5	5.622	146	1.7	0.064	11.422	6.525
13-methyl	2	4.444	30798	222.9	2.510	3.652	3.415
	3	5.192	-	-	3.486	1.832	5.277
	4	5.425	-	-	0.468	2.048	4.996
	5	5.714	10630	127.2	0.054	13.755	6.711
9,10-dimethyl (Cs)	2	4.334	-79196	-	2.713	3.356	5.026
	3	4.997	-	-	2.451	6.318	2.831
	4	5.244	-	-	1.333	2.913	5.416
	5	5.633	462	5.4	0.257	14.007	6.504
9,10-dimethyl (twist)	2	4.300	-147989	-	2.680	1.912	6.098
	3	4.975	-	-	2.909	4.587	4.210
	4	5.144	-	-	1.263	4.114	4.045
	5	5.524	14076	157.4	0.235	6.437	1.952
9,10,13-trimethyl (Cs)	2	4.192	-70112	-	3.238	3.135	5.392
	3	4.884	-	-	2.761	8.647	0.392
	4	5.079	-	-	1.192	1.519	7.016
	5	5.486	5881	64.8	0.319	10.959	2.565
9,10,13-trimethyl (twist)	2	3.983	-43015	-	2.937	1.945	5.728
	3	4.841	-	-	2.528	8.750	2.512
	4	4.952	-	-	1.569	0.899	6.732
	5	5.235	269271	2703.2	0.648	1.889	5.789

Table S5: 1PA and 2PA properties for the higher excited states (S2-S5) in RPSB5 models calculated using TD-DFT/M11.

			**M11**				
Structure	E.S. [no.]	ΔE [eV]	δ^{2PA} [a.u.]	σ^{2PA} [GM]	μ_{01} [D]	$\Delta\mu$ [D]	μ_{11} [D]
9,13-dimethyl	2	4.808	33599	282.4	1.864	2.582	4.556
	3	5.459	2136	23.0	0.105	9.058	3.076
	4	5.477	184	2.0	2.768	3.898	3.070
	5	5.509	4487881	49112.4	0.081	1.231	8.143
9-methyl	2	4.900	13275	115.9	3.369	2.220	5.091
	3	5.305	2359305	24185.0	2.799	0.987	8.035
	4	5.385	8.807	0.1	0.072	9.366	6.421
	5	5.603	1332	15.1	0.028	4.473	2.614
13-methyl	2	4.868	35811	308.5	1.316	2.479	3.931
	3	5.508	403817	4466.6	3.053	-	-
	4	5.562	779	8.7	0.047	9.866	4.285
	5	5.733	53	0.6	0.042	2.428	3.961
9,10-dimethyl (Cs)	2	4.788	26577	221.7	2.182	2.548	4.950
	3	5.266	4.311	0.0	0.037	4.680	2.775
	4	5.423	5589266	59948.1	1.974	1.058	8.380
	5	5.473	557	6.0	0.265	9.781	2.744
9,10-dimethyl (twist)	2	4.765	18931	156.3	2.650	1.822	5.273
	3	5.278	1636578	16557.8	1.485	1.123	5.871
	4	5.348	2677598	27701.3	1.364	2.091	4.946
	5	5.408	78086	824.9	0.288	8.728	2.621
9,10,13-trimethyl (Cs)	2	4.630	37495	292.3	1.445	1.899	5.946
	3	5.162	20	0.2	0.070	4.400	3.412
	4	5.269	3674802	37167.8	3.191	0.602	8.371
	5	5.351	4799	49.6	0.319	8.608	3.720
9,10,13-trimethyl (twist)	2	4.411	31603	223.5	2.337	1.188	5.876
	3	5.205	78235134	771463.1	2.448	0.720	7.648
	4	5.326	133334517	1363819.6	1.181	4.466	2.620
	5	5.401	8397999890	88446150.1	0.312	9.463	3.336

Table S6: 1PA and 2PA properties for the higher excited states (S2-S5) in RPSB5 models calculated using TD-DFT/MN15.

**MN15**							
Structure	E.S. [no.]	ΔE [eV]	δ^{2PA} [a.u.]	σ^{2PA} [GM]	μ_{01} [D]	$\Delta\mu$ [D]	μ_{11} [D]
9,13-dimethyl	2	4.384	1361	9.6	2.313	2.057	4.544
	3	5.027	202635	1867.4	2.794	0.463	6.069
	4	5.202	1895	18.7	0.572	2.010	4.494
	5	5.283	133	1.3	0.018	3.824	2.680
9-methyl	2	4.428	5881	42.2	2.801	0.893	5.854
	3	5.004	156285	1425.1	3.619	0.620	6.373
	4	5.340	78	0.8	1.355	4.752	2.044
	5	5.381	151555	1579.1	0.066	3.615	3.157
13-methyl	2	4.454	1390	10.1	2.320	1.314	4.463
	3	5.108	95557	907.9	2.489	0.377	6.145
	4	5.454	41861	455.0	1.582	1.097	4.777
	5	5.549	25	0.3	0.020	2.135	3.654
9,10-dimethyl (Cs)	2	4.363	1007	7.0	2.329	1.693	5.379
	3	4.987	0	0.0	2.174	4.636	2.575
	4	5.012	169519	1538.1	0.028	1.644	5.421
	5	5.119	11809	112.9	1.069	2.126	4.884
9,10-dimethyl (twist)	2	4.342	1691	11.7	2.225	0.707	5.984
	3	4.933	136527	1208.6	2.554	0.107	6.629
	4	5.045	15560	143.2	1.559	4.562	2.421
	5	5.093	5194	48.4	0.335	3.484	3.410
9,10,13-trimethyl (Cs)	2	4.244	811	5.3	2.976	1.459	5.810
	3	4.869	2	0.0	2.505	4.493	2.768
	4	4.907	250665	2167.7	0.048	2.553	4.705
	5	4.929	79998	708.8	0.696	2.218	5.042
9,10,13-trimethyl (twist)	2	4.067	717	4.3	2.557	0.621	5.991
	3	4.810	519936	4377.5	1.388	0.291	6.311
	4	4.854	376045	3231.0	2.417	4.576	2.382
	5	5.055	18277	166.3	0.500	4.844	1.892

Table S7: 1PA and 2PA properties for the higher excited states (S2-S5) in RPSB5 models calculated using TD-DFT/CAM-B3LYP.

**CAM-B3LYP**							
Structure	E.S. [no.]	ΔE [eV]	δ^{2PA} [a.u.]	σ^{2PA} [GM]	μ_{01} [D]	$\Delta\mu$ [D]	μ_{11} [D]
9,13-dimethyl	2	4.608	4213	32.8	2.112	2.249	4.300
	3	5.168	279000	2732.0	2.694	0.625	5.908
	4	5.406	60900	653.0	1.373	2.921	3.614
	5	5.558	465	5.3	0.013	3.719	2.832
9-methyl	2	4.664	6055	48.2	2.851	1.095	5.613
	3	5.078	32900	1872.0	3.514	0.322	6.557
	4	5.572	336000	22732.2	1.516	4.121	2.793
	5	5.655	1160	13.6	0.059	4.442	2.294
13-methyl	2	4.677	5863	47.0	1.891	1.734	4.142
	3	5.210	100000	994.4	2.654	0.673	5.184
	4	5.665	122000	1436.9	1.855	1.358	4.501
	5	5.806	226	2.8	0.032	2.170	3.706
9,10-dimethyl (Cs)	2	4.582	2429	18.7	2.255	1.950	5.053
	3	5.129	291836	2815.3	2.158	0.446	6.557
	4	5.294	1	0.0	0.029	4.508	2.518
	5	5.335	49332	513.1	1.147	1.549	5.451
9,10-dimethyl (twist)	2	4.567	1608	12.3	2.340	1.035	5.617
	3	5.028	203000	1881.6	2.363	0.848	5.811
	4	5.297	156000	1605.4	1.765	4.639	2.133
	5	5.371	527	5.6	0.120	4.204	2.495
9,10,13-trimethyl (Cs)	2	4.456	3337	24.3	2.511	1.600	5.754
	3	4.999	383869	3517.0	2.260	0.690	6.668
	4	5.183	6453	63.4	1.877	5.617	1.737
	5	5.189	9	0.1	0.053	4.342	2.994
9,10,13-trimethyl (twist)	2	4.262	3024	20.1	2.498	0.806	5.866
	3	4.941	1044735	9298.0	2.065	0.996	5.597
	4	5.125	349679	3374.5	2.171	5.804	0.959
	5	5.345	483193	5064.7	0.493	4.697	1.893

Table S8: 1PA and 2PA properties for the higher excited states (S2-S5) in RPSB5 models calculated using TD-DFT/BHandHLYP.

**BHandHLYP**							
Structure	E.S. [no.]	ΔE [eV]	δ^{2PA} [a.u.]	σ^{2PA} [GM]	μ_{01} [D]	$\Delta\mu$ [D]	μ_{11} [D]
9,13-dimethyl	2	4.643	4433	34.9	2.178	2.067	4.386
	3	5.225	119000	1187.9	2.698	0.337	6.658
	4	5.532	55100	617.3	1.359	2.598	3.813
	5	5.730	1403	16.8	0.087	12.767	6.447
9-methyl	2	4.677	1828	14.7	2.788	0.909	5.594
	3	5.150	78000	757.9	3.634	0.305	6.359
	4	5.697	1020000	12140.9	1.453	3.741	2.883
	5	5.733	105	1.3	0.068	11.403	6.449
13-methyl	2	4.709	6037	49.1	2.047	1.644	3.973
	3	5.265	46200	468.3	2.574	0.446	5.162
	4	5.784	91600	1121.1	1.813	1.302	4.305
	5	5.806	449	5.6	0.041	13.712	8.115
9,10-dimethyl (Cs)	2	4.612	2896	22.6	2.260	1.718	5.098
	3	5.191	126489	1243.4	2.195	0.220	6.592
	4	5.475	47511	520.7	1.141	4.117	2.702
	5	5.760	20	0.2	0.212	14.705	8.117
9,10-dimethyl (twist)	2	4.595	1281	9.9	2.234	0.824	5.637
	3	5.098	87083	830.0	2.523	0.581	5.884
	4	5.443	113167	1225.1	1.660	4.260	2.290
	5	5.719	17895	214.6	0.275	13.500	7.362
9,10,13-trimethyl (Cs)	2	4.498	3848	28.6	2.612	1.610	5.513
	3	5.058	152555	1435.1	1.996	0.569	6.590
	4	5.306	13008	134.3	1.920	5.216	1.935
	5	5.585	118	1.4	0.070	4.365	2.752
9,10,13-trimethyl (twist)	2	4.313	3672	25.0	2.485	0.800	5.692
	3	5.002	405638	3718.5	2.206	0.902	5.495
	4	5.236	131136	1317.8	2.112	5.388	1.116
	5	5.660	1062848	12440.6	0.237	11.441	5.104

Table S9: δ^{2PA} and σ^{2PA} (in a.u. and GM, respectively) for the lowest excited state in RPSB5 models, calculated using two-state model (2SM).

Structure	RI-CC2		M11		MN15		CAM-B3LYP		BHandHLYP	
	δ^{2PA}	σ^{2PA}	δ^{2PA}	σ^{2PA}	δ^{2PA}	σ^{2PA}	δ^{2PA}	σ^{2PA}	δ^{2PA}	σ^{2PA}
9,13-dimethyl	29639	80.3	7158	22.1	2471	7.6	3283	10.5	2698	9.0
9-methyl	25119	65.6	5469	16.2	1726	5.1	2395	7.3	1951	6.3
13-methyl	37398	107.4	9311	30.5	3566	11.6	4692	15.7	3939	13.9
9,10-dimethyl (Cs)	21199	57.8	4872	14.7	1451	4.5	2006	6.3	1640	5.4
9,10-dimethyl (twist)	16209	42.7	3587	10.5	951	2.8	1341	4.1	1023	3.3
9,10,13-trimethyl (Cs)	43253	107.1	11909	34.0	4366	12.4	5887	17.3	5060	15.7
9,10,13-trimethyl (twist)	23316	53.8	6766	17.7	2095	5.5	3053	8.3	2543	7.3

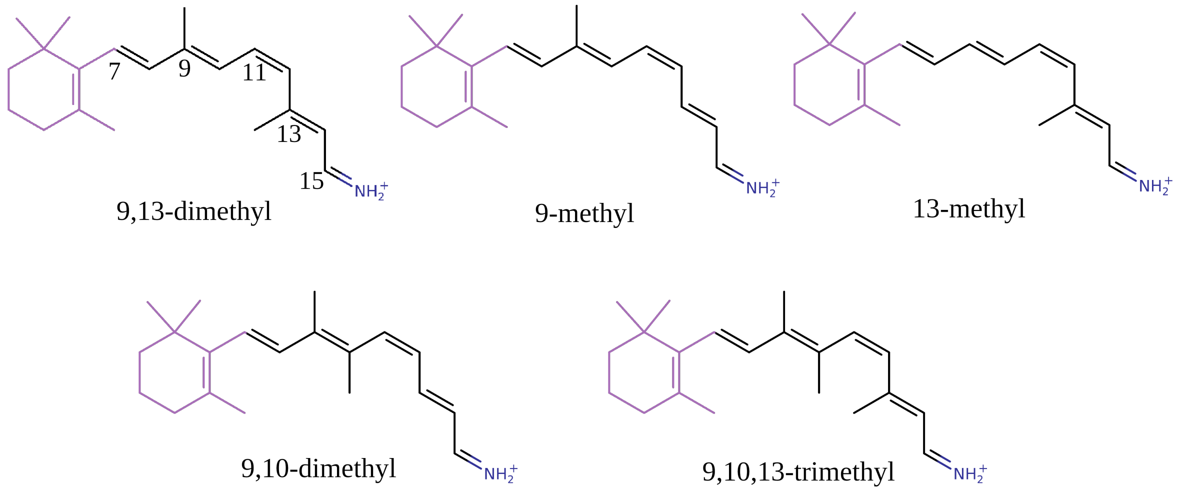

Figure S5: Structures of the native RPSB and its (de)methylated analogues. The β-ionon ring shown in pink is missing in RPSB5 models.

Table S10: 1PA energies (ΔE), two-photon transition strengths (δ^{2PA}), 2PA cross-sections (σ^{2PA}), S0-S1 transition dipole moments (μ_{01}), the first excited state (μ_{11}) and the ground state (μ_{00}) permanent electric dipole moments, and their differences ($\Delta\mu$) for RPSB chromophores (β-ionone ring inclusive) calculated using TD-DFT with the M11 functional and aug-cc-pVDZ basis set based on M06-2X/cc-pVDZ geometries. The table provides values with current-density response: (A) included, (B) excluded ('curswitchdisengage' keyword in the input file; gauge variant) in the M11 calculations.

			**A**				
Structure	ΔE [eV]	δ^{2PA} [a.u.]	σ^{2PA} [GM]	μ_{01} [D]	$\Delta\mu$ [D]	μ_{00} [D]	μ_{11} [D]
9,13-dimethyl	2.576	46369	112.8	12.846	6.799	17.852	11.171
9-methyl	2.558	35052	84.0	12.145	6.222	17.403	11.416
13-methyl	2.605	58119	144.5	13.584	7.158	18.210	11.157
9,10-dimethyl	2.511	31289	72.2	12.863	5.410	16.934	11.720
9,10,13-trimethyl	2.417	41659	89.1	12.532	5.988	16.951	11.084
			**B**				
Structure	ΔE [eV]	δ^{2PA} [a.u.]	σ^{2PA} [GM]	μ_{01} [D]	$\Delta\mu$ [D]	μ_{00} [D]	μ_{11} [D]
9,13-dimethyl	2.564	46084	111.0	12.827	6.799	17.852	11.171
9-methyl	2.545	34837	82.7	12.125	6.222	17.403	11.416
13-methyl	2.592	57791	142.2	13.563	7.158	18.210	11.157
9,10-dimethyl	2.498	31090	71.1	12.840	5.410	16.934	11.720
9,10,13-trimethyl	2.404	41388	87.6	12.507	5.988	16.951	11.084

Table S11: Cartesian coordinates of RPSB and its (de)methylated analogues (see Fig. S5 for the structures) calculated at the M06-2X/cc-pVDZ level of theory.

RPSB (9,13-dimethyl - final Energy: -873.639457434 a.u.).

C	-4.746182	-2.396829	-0.431095
C	-3.581825	-1.458905	-0.646485
C	-3.556941	-0.210110	-0.111966
C	-4.719923	0.379102	0.705753
C	-5.649099	-0.743049	1.192564
C	-6.009056	-1.720687	0.085561
C	-2.492807	-2.045362	-1.506074
C	-2.444689	0.717858	-0.311801
C	-1.118233	0.433098	-0.267589
C	-0.068019	1.411628	-0.438449
C	1.236032	0.970563	-0.310969
C	2.380553	1.796604	-0.448501
C	3.732959	1.526992	-0.339873
C	4.412499	0.317199	-0.031999
C	3.656634	-0.947137	0.246066
C	-5.504075	1.382645	-0.155871
C	-4.176265	1.105185	1.946348
C	5.809022	0.371988	-0.000514
C	6.648228	-0.705175	0.282276
C	-0.456377	2.831733	-0.729852
C	8.863425	-1.736996	0.596552
N	7.965146	-0.631201	0.305163
H	-1.839152	-1.289383	-1.954866
H	-1.867842	-2.738889	-0.918052
H	-2.941057	-2.641421	-2.313794
H	-4.412332	-3.172591	0.283946
H	-4.947376	-2.933195	-1.371108
H	-6.718418	-2.476604	0.449422
H	-6.508442	-1.190913	-0.740317
H	-6.551974	-0.288331	1.628803
H	-5.142858	-1.292911	2.004810
H	-4.860258	2.198461	-0.518888
H	-5.953672	0.899246	-1.034094
H	-6.308181	1.841334	0.439707
H	-3.586535	1.996522	1.683371
H	-5.013494	1.437931	2.578275
H	-3.539163	0.437213	2.545372
H	-2.734279	1.765797	-0.440713
H	-0.799302	-0.589339	-0.053525
H	1.353661	-0.087905	-0.092381
H	2.174564	2.841795	-0.687828
H	4.387136	2.383348	-0.514683
H	6.282279	1.333539	-0.214732
H	6.246816	-1.695957	0.505737
H	4.298213	-1.797496	0.493002
H	2.965472	-0.788083	1.085655
H	3.054829	-1.219369	-0.632666
H	8.391433	0.270587	0.102959
H	9.525330	-1.923767	-0.259326
H	9.472936	-1.507692	1.480507
H	8.268296	-2.635769	0.793648
H	-1.068786	2.873009	-1.641575
H	0.394201	3.505025	-0.860493
H	-1.073252	3.222157	0.093197

RPSB (9-methyl - final Energy: -834.344191977 a.u.).

C	5.503147	1.954085	-0.035026
C	4.191535	2.500730	-0.581918
C	3.100112	1.467290	-0.730552
C	3.170956	0.254824	-0.120418
C	4.377701	-0.193668	0.723304
C	5.221459	1.023099	1.132814
C	1.967674	1.916970	-1.616125
C	2.132851	-0.764463	-0.258991
C	0.787697	-0.579660	-0.237455
C	-0.180264	-1.645404	-0.353296
C	0.314011	-3.048174	-0.553990
C	5.232625	-1.187030	-0.080922
C	3.894048	-0.879972	2.010503
C	-1.516992	-1.307733	-0.265248
C	-2.595903	-2.215256	-0.358497
C	-3.950306	-1.931689	-0.266130
C	-4.490709	-0.655048	-0.045979
C	-5.848101	-0.388856	0.046478
C	-6.293604	0.915571	0.271530
N	-7.553899	1.282113	0.377678
C	-8.016528	2.641201	0.613068
H	1.378356	1.087452	-2.022252
H	1.287401	2.586783	-1.063020
H	2.366325	2.504396	-2.455414
H	3.802440	3.291800	0.087019
H	4.348639	2.991962	-1.554387
H	6.154950	2.782021	0.275850
H	6.038112	1.413050	-0.830756
H	6.157316	0.665054	1.589020
H	4.678023	1.582710	1.913777
H	4.650881	-2.069227	-0.389318
H	5.643214	-0.726340	-0.989885
H	6.069887	-1.547482	0.536105
H	3.373728	-1.828525	1.808265
H	4.755947	-1.108004	2.655565
H	3.209585	-0.226219	2.571727
H	2.500925	-1.793795	-0.319238
H	0.388466	0.425887	-0.089275
H	-2.352071	-3.266165	-0.524579
H	-4.650077	-2.762891	-0.368884
H	-6.573402	-1.199651	-0.055432
H	-5.561161	1.723183	0.373130
H	-8.264986	0.559129	0.289160
H	-8.654465	2.972580	-0.216614
H	-8.587341	2.689462	1.549556
H	-7.147028	3.303982	0.686326
H	0.931703	-3.102472	-1.461442
H	-0.486510	-3.786916	-0.641713
H	0.953227	-3.339993	0.292399
H	-1.738049	-0.249123	-0.111720
H	-3.805214	0.191654	0.063395

RPSB (13-methyl - final Energy: -834.342104417 a.u.).

C	3.884533	1.422187	-0.317541
C	3.688414	0.093684	-0.094934
C	4.797821	-0.847155	0.409165
C	5.946795	-0.032525	1.022569
C	6.375465	1.126022	0.137294
C	5.210607	2.089435	-0.044158
C	2.423956	-0.573944	-0.374449
C	1.155814	-0.110704	-0.190378
C	0.026532	-0.927075	-0.497141
C	-1.282735	-0.559521	-0.312580
C	-2.321359	-1.468118	-0.650987
C	-3.698504	-1.390468	-0.577797
C	-4.529163	-0.332668	-0.118677
C	-5.909060	-0.547758	-0.164636
C	-6.877041	0.370813	0.240778
C	-9.206179	1.087206	0.603865
C	5.317540	-1.708145	-0.753663
C	4.246713	-1.770351	1.507581
C	2.838460	2.361356	-0.857979
C	-3.933507	0.946069	0.388207
N	-8.176025	0.148325	0.189044
H	0.232451	-1.922554	-0.907880
H	2.076923	1.855147	-1.462537
H	2.328632	2.888984	-0.034112
H	3.315669	3.134407	-1.475746
H	5.081350	2.705504	0.866202
H	5.417897	2.806142	-0.853498
H	7.234030	1.653568	0.574760
H	6.701980	0.752314	-0.845452
H	6.790288	-0.710797	1.223757
H	5.616341	0.365360	1.997671
H	4.512307	-2.301902	-1.212761
H	5.768149	-1.093245	-1.544877
H	6.076847	-2.415556	-0.386771
H	3.492325	-2.473901	1.124960
H	5.066685	-2.367255	1.934556
H	3.790456	-1.185490	2.320352
H	2.507752	-1.610413	-0.723431
H	0.976185	0.878386	0.234563
H	-1.485490	0.429229	0.093398
H	-1.953031	-2.419844	-1.049689
H	-4.231819	-2.277674	-0.924281
H	-6.258683	-1.509646	-0.548058
H	-6.600987	1.351753	0.633618
H	-4.676421	1.678602	0.715662
H	-3.269192	0.732827	1.237662
H	-3.325018	1.408880	-0.401693
H	-8.487236	-0.751841	-0.169771
H	-9.860908	1.330273	-0.243318
H	-9.809112	0.655454	1.413516
H	-8.726821	2.004973	0.962645

RPSB (9,10-dimethyl - final Energy: -873.633513180 a.u.).

C	-5.400828	-0.809399	1.355087
C	-5.893588	-1.649483	0.188302
C	-4.707623	-2.293951	-0.516647
C	-3.530166	-1.372847	-0.730163
C	-3.415020	-0.188381	-0.070920
C	-4.481945	0.336698	0.906072
C	-2.536008	-1.896552	-1.734429
C	-2.297678	0.728136	-0.277286
C	-0.986538	0.398564	-0.435397
C	0.086709	1.356162	-0.570770
C	1.405559	0.902850	-0.656670
C	2.471203	1.838694	-0.560384
C	3.840064	1.662577	-0.395325
C	4.548409	0.487679	-0.093152
C	5.925927	0.454607	0.085753
C	6.559723	-0.741291	0.421999
N	7.855818	-0.891893	0.611792
C	8.512866	-2.141049	0.962364
C	-5.305087	1.445934	0.230693
C	-3.807414	0.910427	2.162436
C	-0.301033	2.811571	-0.553646
C	1.712906	-0.564594	-0.840852
H	4.007437	-0.449065	0.051179
H	-1.942329	-1.105133	-2.207162
H	-1.841694	-2.609573	-1.257585
H	-3.062142	-2.454371	-2.521513
H	-4.340841	-3.158104	0.069560
H	-5.010888	-2.710129	-1.489582
H	-6.591333	-2.424520	0.533736
H	-6.449056	-1.020168	-0.523982
H	-6.244005	-0.383953	1.920877
H	-4.841821	-1.457434	2.052200
H	-4.669054	2.281604	-0.099479
H	-5.844713	1.072131	-0.650266
H	-6.038993	1.855163	0.941688
H	-3.214482	1.811744	1.944537
H	-4.574677	1.191483	2.899500
H	-3.142970	0.166215	2.626740
H	2.187412	2.889177	-0.609731
H	4.440202	2.574583	-0.441433
H	6.516783	1.365688	-0.036345
H	5.961172	-1.649754	0.546370
H	-0.722078	-0.653810	-0.368391
H	8.447100	-0.070589	0.505339
H	-2.561471	1.788507	-0.229591
H	7.756137	-2.929781	1.040718
H	9.241681	-2.416656	0.188889
H	9.029630	-2.040672	1.925841
H	0.494309	3.494809	-0.856789
H	-1.141116	2.972892	-1.241227
H	-0.641523	3.102823	0.451777
H	2.613016	-0.678518	-1.459294
H	1.881564	-1.085331	0.115858
H	0.905394	-1.084117	-1.367361

RPSB (9,10,13-trimethyl - final Energy: -912.927710001 a.u.).

C	-6.232833	-1.411213	0.162231
C	-5.128051	-2.072009	-0.650807
C	-3.888980	-1.226249	-0.824967
C	-3.654732	-0.127620	-0.058097
C	-4.639556	0.375119	1.012434
C	-5.633741	-0.735209	1.384665
C	-2.977855	-1.720659	-1.918623
C	-2.473985	0.716543	-0.221838
C	-1.199723	0.310264	-0.468229
C	-0.076389	1.207177	-0.618038
C	-0.359134	2.682254	-0.534940
C	-5.392522	1.609111	0.488448
C	-3.875657	0.761946	2.288771
C	1.202330	0.682592	-0.780664
C	2.311670	1.559070	-1.005132
C	3.672970	1.342049	-0.884112
C	4.373095	0.412575	-0.070798
C	5.754873	0.293927	-0.261975
C	6.563127	-0.539872	0.505831
N	7.859847	-0.709237	0.321805
C	8.712635	-1.580450	1.113660
C	3.688797	-0.320024	1.052533
H	-2.332594	-0.936167	-2.330972
H	-2.333323	-2.535779	-1.547545
H	-3.576451	-2.145613	-2.736402
H	-4.812288	-3.015537	-0.166153
H	-5.497671	-2.365304	-1.645343
H	-6.980515	-2.159714	0.458422
H	-6.757499	-0.670783	-0.461087
H	-6.419524	-0.304749	2.024521
H	-5.106638	-1.492776	1.990217
H	-4.702219	2.421621	0.214383
H	-5.993355	1.370989	-0.399995
H	-6.063568	1.999499	1.268823
H	-3.218391	1.631337	2.135347
H	-4.590092	1.024831	3.083441
H	-3.258483	-0.075985	2.646519
H	-2.648477	1.785210	-0.063128
H	-1.005736	-0.758855	-0.520284
C	1.406576	-0.816209	-0.869047
H	2.065915	2.532618	-1.435351
H	4.309034	2.050129	-1.421638
H	6.217796	0.863525	-1.071378
H	6.137591	-1.123772	1.326682
H	4.260978	-0.201891	1.983030
H	2.684582	0.078180	1.227790
H	3.603180	-1.395727	0.840662
H	8.300989	-0.190519	-0.434220
H	9.169525	-2.348606	0.475810
H	9.506034	-0.997172	1.599414
H	8.104123	-2.068514	1.883371
H	-1.118385	2.966810	-1.276355
H	0.521387	3.313491	-0.673269
H	-0.770319	2.921987	0.457552
H	0.735525	-1.238711	-1.630431
H	1.201189	-1.338397	0.076668
H	2.431265	-1.049661	-1.179568
